# Six plant extracts delay yeast chronological aging through different signaling pathways

**DOI:** 10.18632/oncotarget.10689

**Published:** 2016-07-18

**Authors:** Vicky Lutchman, Pamela Dakik, Mélissa McAuley, Berly Cortes, George Ferraye, Leonid Gontmacher, David Graziano, Fatima-Zohra Moukhariq, Éric Simard, Vladimir I. Titorenko

**Affiliations:** ^1^ Department of Biology, Concordia University, Montreal, Quebec, Canada; ^2^ Idunn Technologies Inc., Rosemere, Quebec, Canada

**Keywords:** yeast, cellular aging, longevity, plant extracts, aging-delaying chemical compounds, Gerotarget

## Abstract

Our recent study has revealed six plant extracts that slow yeast chronological aging more efficiently than any chemical compound yet described. The rate of aging in yeast is controlled by an evolutionarily conserved network of integrated signaling pathways and protein kinases. Here, we assessed how single-gene-deletion mutations eliminating each of these pathways and kinases affect the aging-delaying efficiencies of the six plant extracts. Our findings imply that these extracts slow aging in the following ways: 1) plant extract 4 decreases the efficiency with which the pro-aging TORC1 pathway inhibits the anti-aging SNF1 pathway; 2) plant extract 5 mitigates two different branches of the pro-aging PKA pathway; 3) plant extract 6 coordinates processes that are not assimilated into the network of presently known signaling pathways/protein kinases; 4) plant extract 8 diminishes the inhibitory action of PKA on SNF1; 5) plant extract 12 intensifies the anti-aging protein kinase Rim15; and 6) plant extract 21 inhibits a form of the pro-aging protein kinase Sch9 that is activated by the pro-aging PKH1/2 pathway.

## INTRODUCTION

**Figure 1:**
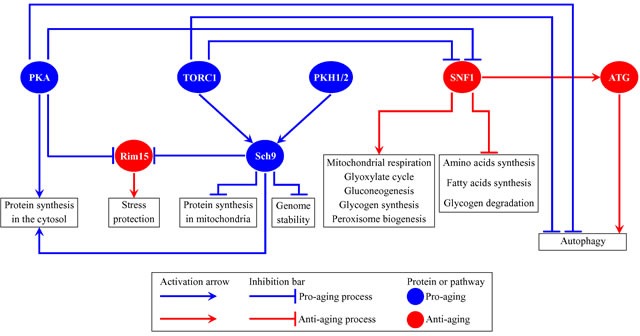
Several nutrient- and energy-sensing signaling pathways and protein kinases converge into a network which defines the rate of yeast chronological aging This network coordinates longevity-defining cellular processes named in boxes. Activation arrows and inhibition bars denote pro-aging processes (displayed in blue color) or anti-aging processes (displayed in red color). Pro-aging or anti-aging signaling pathways and protein kinases are displayed in blue or red color, respectively. Please see text for additional details. Abbreviations: ATG, autophagy; PKA, protein kinase A; PKH1/2, Pkb-activating kinase homologs 1 and 2; Rim15, an anti-aging protein kinase; Sch9, a pro-aging protein kinase; SNF1, sucrose non-fermenting protein 1; TORC1, target of rapamycin complex 1.

The budding yeast *Saccharomyces cerevisiae* is a beneficial model organism for the discovery of genes, signaling pathways and chemical compounds that slow cellular and organismal aging in eukaryotes across phyla [1–15]. Studies in *S. cerevisiae* have demonstrated that the major aspects of biological aging are evolutionarily conserved [1, 4–6, 10–13, 16–18]. One of these aspects is the convergence of certain signaling pathways and protein kinases into a network that controls the rate of aging [1, 6, 11, 19–31]. In chronologically aging yeast, this network integrates the following: 1) the pro-aging TORC1 (target of rapamycin complex 1) pathway [32–35]; 2) the pro-aging PKA (protein kinase A) pathway [36–40]; 3) the pro-aging PKH1/2 (Pkb-activating kinase homolog) pathway [30, 41–44]; 4) the anti-aging SNF1 (sucrose non-fermenting) pathway [45–50]; 5) the anti-aging ATG (autophagy) pathway [5, 51–58]; 6) the pro-aging protein kinase Sch9, which is stimulated by the TORC1 and PKH1/2 pathways [11, 40, 42–44, 59, 60]; and 7) the anti-aging protein kinase Rim15, which is inhibited by the TORC1, PKA and PKH1/2 pathways [11, 37, 40, 59, 61, 62] (Figure 1). This network of signaling pathways and protein kinases coordinates certain longevity-defining cellular processes, including stress responses, protein synthesis in the cytosol and mitochondria, maintenance of nuclear and mitochondrial genomes, autophagy, mitochondrial respiration, peroxisome biogenesis, gluconeogenesis, lipid metabolism, glyoxylate cycle, glycogen synthesis and degradation, and the synthesis of amino acids and fatty acids [1, 6, 11, 27–32, 59, 61–73] (Figure 1). Information flow along this network in yeast is controlled by such aging-delaying chemical compounds as resveratrol, rapamycin, caffeine, spermidine, myriocin, methionine sulfoxide, lithocholic acid and cryptotanshinone [3, 5, 6, 8–10, 12, 14, 43, 54, 74–78].

We have recently discovered six plant extracts (PEs) that increase yeast chronological lifespan (CLS) to a greater extent than any of the presently known longevity-extending chemical compounds [78]. We demonstrated that each of these PEs (which we call PE4, PE5, PE6, PE8, PE12 and PE21) decelerates chronological aging and has different effects on certain longevity-defining cellular processes [78]. In this study, we investigated how a mutational impairment of each of the signaling pathways and protein kinases comprising the longevity-defining network influences the aging-delaying efficiencies of PE4, PE5, PE6, PE8, PE12 and PE21. We show that PE4, PE5, PE8, PE12 and PE21 delay yeast chronological aging through different signaling pathways and protein kinases converging into this network. In contrast, PE6 slows aging by coordinating processes that are not integrated into the network.

## RESULTS

### The rationale of our experimental approach

PE4, PE5, PE6, PE8, PE12 and PE21 may have different effects on signaling pathways and/or protein kinases integrated into the longevity-defining network. To identify the pathways and kinases through which each of these PEs slows yeast chronological aging, we assessed such effects. Specifically, we elucidated how mutations eliminating these signaling pathways and protein kinases affect the efficiency with which each of the six PEs extends yeast CLS. Table 1 shows the single-gene-deletion mutations used in this study. This table also demonstrates how each of the mutations impacts different longevity-defining signaling pathways and protein kinases, and how it alters yeast CLS. We investigated the effects of the following single-gene-deletion mutations shown in Table 1: 1) *tor1Δ*, which impairs the pro-aging TORC1 pathway and increases CLS [32, 35]; 2) *ras2Δ*, which weakens the pro-aging PKA pathway and extends CLS [39]; 3) *rim15Δ*, which eliminates the anti-aging protein kinase Rim15 and shortens CLS [37]; 4) *sch9Δ*, which removes the pro-aging protein kinase Sch9 and increases CLS [37]; 5) *pkh2Δ*, which weakens the pro-aging PKH1/2 pathway and extends CLS [41, 42]; 6) *snf1Δ*, which impairs the anti-aging SNF1 pathway and decreases CLS [50, 62]; and 7) *atg1Δ*, which deteriorates the anti-aging ATG pathway and shortens CLS [51, 52].

**Table 1:** Single-gene-deletion mutations used in this study and their known effects on longevity-defining signaling pathways and longevity of chronologically aging yeast

Single-gene-deletion mutation	Protein eliminated	Longevity-defining signaling pathway(s) affected	Effect on longevity
*tor1Δ*	Tor1	TORC1	⇧Extended [32, 35]
*ras2Δ*	Ras2	PKA	⇧Extended [39]
*rim15Δ*	Rim15	TORC1, PKA, PKH1/2	⇩Shortened [37]
*sch9Δ*	Sch9	TORC1, PKA	⇧Extended [37]
*pkh2Δ*	Pkh2	PKH1/2	⇧Extended [41, 42]
*snf1Δ*	Snf1	SNF1	⇩Shortened [50, 62]
*atg1Δ*	Atg1	ATG	⇩Shortened [51, 52]

**Figure 2:**
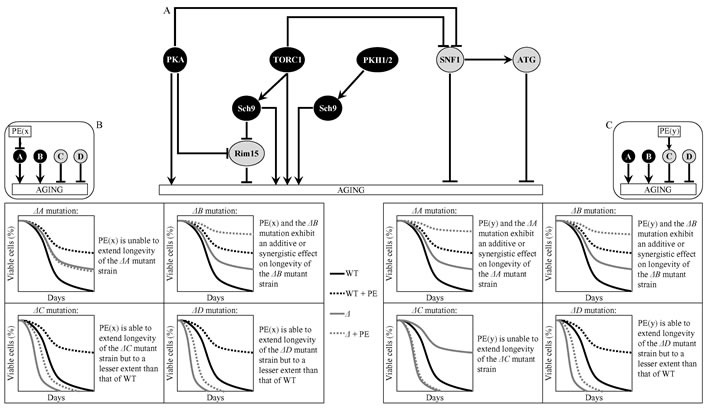
A logical framework for identifying signaling pathways and/or protein kinases controlled by the longevity-extending PE(x) and PE(y) **A.** A schematic depiction of a network which integrates several signaling pathways and protein kinases to define the rate of yeast chronological aging. Pro-aging signaling pathways or protein kinases A and B are shown in black color. Anti-aging signaling pathways or protein kinases C and D are displayed in grey color. Abbreviations are as in Figure 1. **B.** Predicted effect of PE(x), which extends yeast CLS by inhibiting a pro-aging pathway/protein kinase A, on longevity of the *ΔA*, *ΔB*, *ΔC* or *ΔD* mutant strain lacking a signaling pathway/protein kinase A, B, C or D. **C.** Predicted effect of PE(y), which extends yeast CLS by activating an anti-aging pathway/protein kinase C, on longevity of the *ΔA*, *ΔB*, *ΔC* or *ΔD* mutant strain lacking the corresponding signaling pathway/protein kinase. Abbreviation: WT, wild-type strain.

A logical framework for identifying signaling pathways and/or protein kinases controlled by each of the six longevity-extending PEs is schematically depicted in Figure 2. Pro-aging signaling pathways or protein kinases A and B in this figure are displayed in black color, whereas their anti-aging counterparts C and D are shown in grey color. One could envision that if PE(x) extends yeast CLS by inhibiting a pro-aging pathway/protein kinase A, this PE: 1) is unable to extend longevity of the *ΔA* mutant strain lacking this signaling pathway/protein kinase (Figure 2B); 2) exhibits an additive or synergistic longevity-extending effect with the *ΔB* mutation, which eliminates the pro-aging signaling pathway/protein kinase B (Figure 2B); and 3) is able to prolong longevity of the *ΔC* or *ΔD* mutant strain, which lacks the anti-aging signaling pathway/protein kinase C or D (respectively), but to a lesser extent than that of wild-type (WT) strain (Figure 2B). It is also plausible that that if PE(y) extends yeast CLS by activating an anti-aging pathway/protein kinase C, this PE: 1) displays an additive or synergistic longevity-extending effect with the *ΔA* or *ΔB* mutation, which eliminates the pro-aging signaling pathway/protein kinase A or B (respectively) (Figure 2C); 2) is incapable of increasing longevity of the *ΔC* mutant strain deficient in the anti-aging signaling pathway/protein kinase C (Figure 2C); and 3) can extend longevity of the *ΔD* mutant strain, which lacks the anti-aging signaling pathway/protein kinase D, although not as considerably as that of WT strain (Figure 2C).

### PE4 delays chronological aging by attenuating the inhibitory effect of TORC1 on SNF1

**Figure 3:**
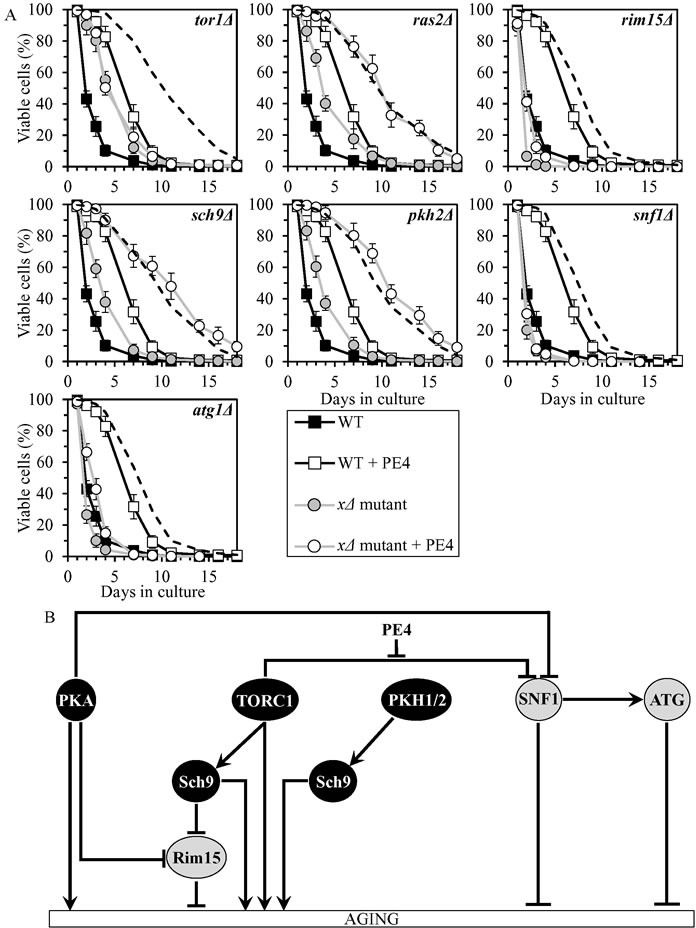
PE4 extends yeast CLS by weakening the restraining action of TORC1 on SNF1 **A.** Cells of the wild-type (WT) and indicated mutant strains were grown in the synthetic minimal YNB medium (0.67% Yeast Nitrogen Base without amino acids) initially containing 2% glucose, in the presence of 0.5% PE4 (ethanol was used as a vehicle at the final concentration of 2.5%) or in its absence (cells were subjected to ethanol-mock treatment). Survival curves of chronologically aging WT and mutant strains cultured with or without 0.5% PE4 are shown. Data are presented as means ± SEM (*n* = 7). The dotted line indicates the predicted survival curve of a particular mutant strain cultured with PE4 if this PE exhibits an additive longevity-extending effect with the mutation. Data for the mock-treated WT strain are replicated in all graphs of this Figure and in all graphs of Figure 4. Data for each of the mock-treated mutant strains presented in this Figure are replicated in the corresponding graphs of Figure 4. Data for the WT strain cultured with PE4 are replicated in all graphs of this Figure. **B.** The effect of PE4 on the signaling pathways and protein kinases comprising the longevity-defining network. This effect is inferred from the data presented in **A.**, Tables 2 and 3, and Suppl. Figs. S1 and S7. Abbreviations: as in the legend to Figure 1.

PE4 exhibited additive longevity-extending effects with the *ras2Δ*, *sch9Δ* and *pkh2Δ* mutations, which eliminate different signaling pathways/protein kinases comprising the longevity-defining network (Figure 3A, Tables 2 and 3, Suppl. Figure S1; note that data for the mock-treated WT strain and for the WT strain cultured with PE4 are replicated in all graphs of Figure 3A and Suppl. Figure S1). PE4 caused a decrease in slope of the Gompertz mortality rate (also known as mortality rate coefficient α) and an increase in the mortality rate doubling time (MRDT) for *ras2Δ*, *sch9Δ* and *pkh2Δ* (Suppl. Figure S7; note that data for the mock-treated WT strain and for the WT strain cultured with PE4 are replicated in all graphs of this figure). Such changes in the values of α and MRDT are characteristic of interventions that decrease the rate of biological aging [79–83]. Thus, PE4 delays yeast chronological aging independently of the pro-aging PKA pathway, the pro-aging PKH1/2 pathway or the pro-aging protein kinase Sch9.

PE4 extended longevities of the *rim15Δ* and *atg1Δ* mutant strains, although to a lesser extent than that of WT strain (Figure 3A, Tables 2 and 3, Suppl. Figure S1). PE4 reduced the value of α and elevated the value of MRDT for *rim15Δ* and *atg1Δ*, though not as significantly as for WT (Suppl. Figure S7). Hence, PE4 slows yeast chronological aging not through the anti-aging protein kinase Rim15 or the anti-aging ATG pathway.

PE4 was unable to extend the CLS of *tor1Δ* and *snf1Δ* (Figure 3A, Tables 2 and 3, Suppl. Figure S1) and did not alter the values of α or MRDT for these mutant strains (Suppl. Figure S7). We concluded that PE4 delays yeast chronological aging *via* the pro-aging TORC1 pathway and the anti-aging SNF1 pathway, by weakening the known [61, 66] inhibitory effect of TORC1 on SNF1 (Figure 3B).

**Table 2:** *p* Values for pairs of survival curves of a yeast strain cultured with or without the indicated plant extract (PE)

Strain without PE	Same strain with the indicated PE					
	PE4	PE5	PE6	PE8	PE12	PE21
WT	< 0.0001	< 0.0001	< 0.0001	< 0.0001	< 0.0001	< 0.0001
*tor1Δ*	0.8899	< 0.0001	< 0.0001	< 0.0001	< 0.0001	< 0.0001
*ras2Δ*	< 0.0001	0.3664	< 0.0001	0.41888	< 0.0001	< 0.0001
*rim15Δ*	0.0042	0.0168	< 0.0001	0.0184	0.6453	< 0.0001
*sch9Δ*	< 0.0001	< 0.0001	< 0.0001	0.0006	< 0.0001	0.0306
*pkh2Δ*	< 0.0001	< 0.0001	< 0.0001	< 0.0001	< 0.0001	< 0.0001
*snf1Δ*	0.5873	0.0075	< 0.0001	0.7124	0.0132	< 0.0001
*atg1Δ*	0.0027	0.0061	< 0.0001	0.0086	0.0208	< 0.0001

**Figure 4:**
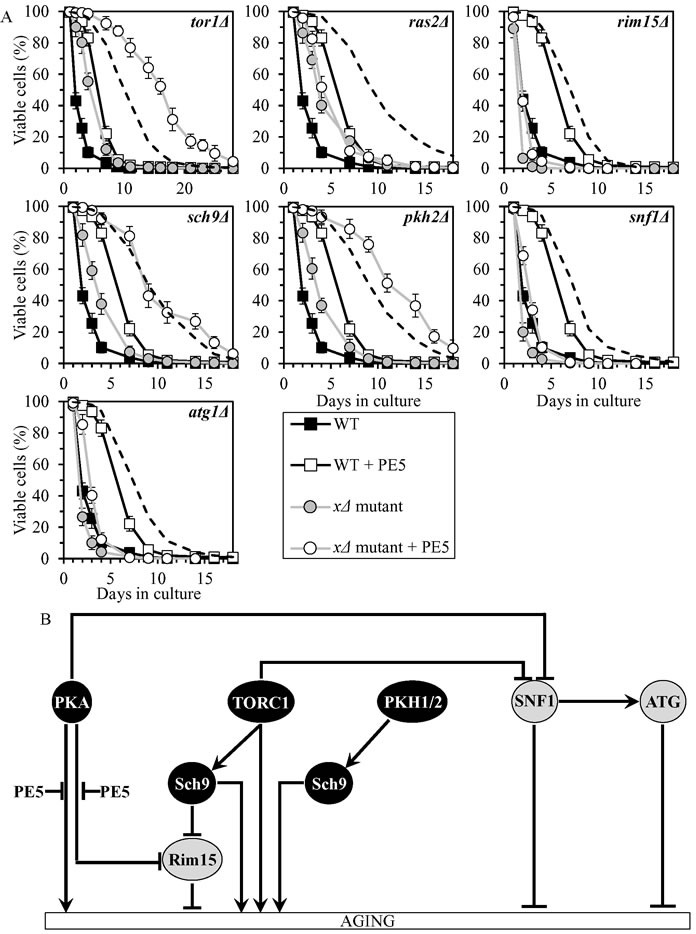
PE5 extends yeast CLS by weakening two branches of the PKA signaling pathway **A.** Cells of the wild-type (WT) and indicated mutant strains were grown in the synthetic minimal YNB medium (0.67% Yeast Nitrogen Base without amino acids) initially containing 2% glucose, in the presence of 0.5% PE5 (ethanol was used as a vehicle at the final concentration of 2.5%) or in its absence (cells were subjected to ethanol-mock treatment). Survival curves of chronologically aging WT and mutant strains cultured with or without 0.5% PE5 are shown. Data are presented as means ± SEM (*n* = 7). The dotted line indicates the predicted survival curve of a particular mutant strain cultured with PE5 if this PE exhibits an additive longevity-extending effect with the mutation. Data for the mock-treated WT strain are replicated in all graphs of this Figure and in all graphs of Figure 3. Data for each of the mock-treated mutant strains presented in this Figure are replicated in the corresponding graphs of Figure 3. Data for the WT strain cultured with PE5 are replicated in all graphs of this Figure. **B.** The effect of PE5 on the signaling pathways and protein kinases comprising the longevity-defining network. This effect is inferred from the data presented in **A.**, Tables 2 and 3, and Suppl. Figs. S2 and S8. Abbreviations: as in the legend to Figure 1.

**Figure 5:**
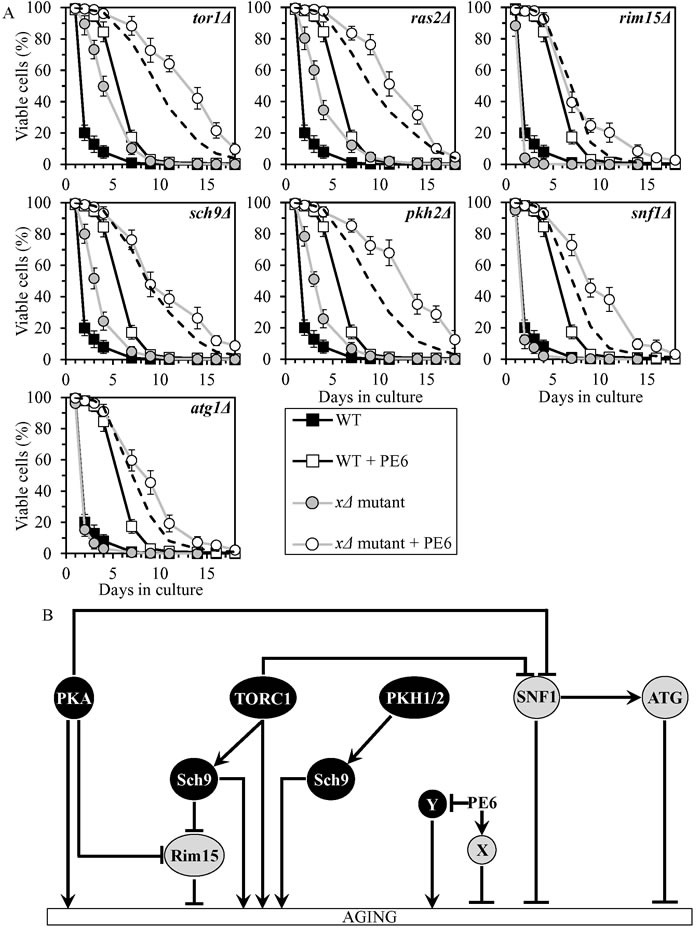
PE6 extends yeast CLS independently of presently known longevity-defining signaling pathways/protein kinases **A.** Cells of the wild-type (WT) and indicated mutant strains were grown in the synthetic minimal YNB medium (0.67% Yeast Nitrogen Base without amino acids) initially containing 2% glucose, in the presence of 1.0% PE6 (ethanol was used as a vehicle at the final concentration of 5.0%) or in its absence (cells were subjected to ethanol-mock treatment). Survival curves of chronologically aging WT and mutant strains cultured with or without 1.0% PE6 are shown. Data are presented as means ± SEM (*n* = 8). The dotted line indicates the predicted survival curve of a particular mutant strain cultured with PE6 if this PE exhibits an additive longevity-extending effect with the mutation. Data for the mock-treated WT strain and for the WT strain cultured with PE6 are replicated in all graphs of this Figure. **B.** The effects of PE6 on anti-aging and/or pro-aging processes that are not controlled by the network of presently known signaling pathways/protein kinases. These effects are inferred from the data presented in **A.**, Tables 2 and 3, and Suppl. Figs. S3 and S9. Abbreviations: as in the legend to Figure 1.

**Figure 6:**
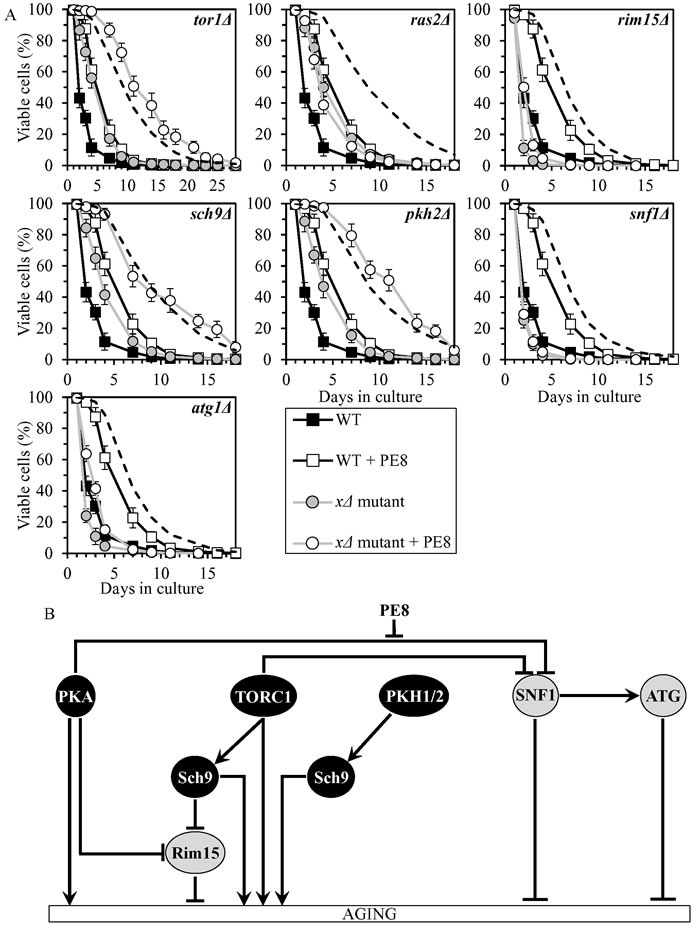
PE8 extends yeast CLS by attenuating the inhibitory effect of PKA on SNF1 **A.** Cells of the wild-type (WT) and indicated mutant strains were grown in the synthetic minimal YNB medium (0.67% Yeast Nitrogen Base without amino acids) initially containing 2% glucose, in the presence of 0.3% PE8 (ethanol was used as a vehicle at the final concentration of 1.5%) or in its absence (cells were subjected to ethanol-mock treatment). Survival curves of chronologically aging WT and mutant strains cultured with or without 0.3% PE8 are shown. Data are presented as means ± SEM (*n* = 6). The dotted line indicates the predicted survival curve of a particular mutant strain cultured with PE8 if this PE exhibits an additive longevity-extending effect with the mutation. Data for the mock-treated WT strain and for the WT strain cultured with PE8 are replicated in all graphs of this Figure. **B.** The effect of PE8 on the signaling pathways and protein kinases integrated into the longevity-defining network. This effect is inferred from the data presented in **A.**, Tables 2 and 3, and Suppl. Figs. S4 and S10. Abbreviations: as in the legend to Figure 1.

**Figure 7:**
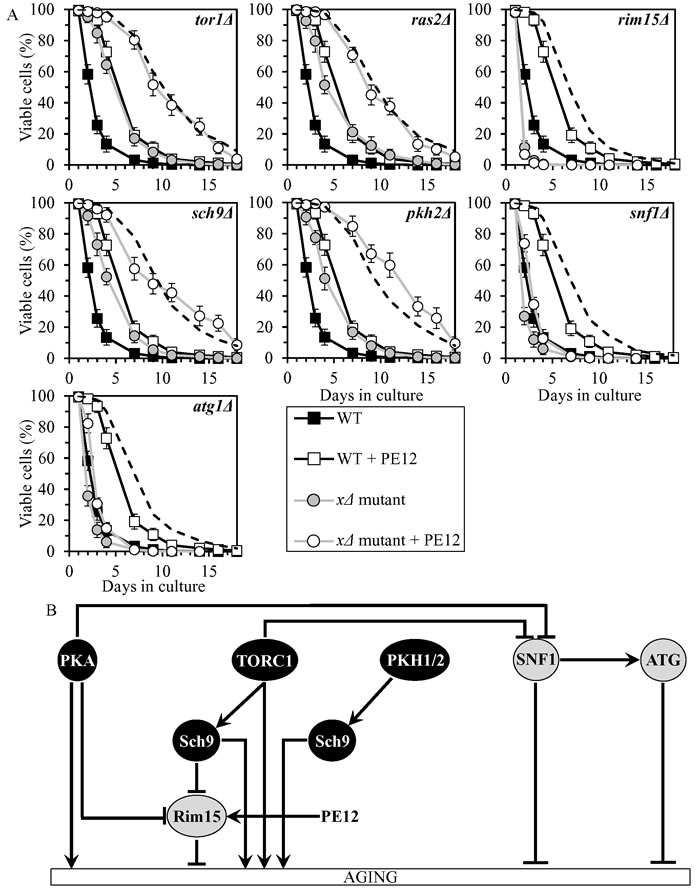
PE12 extends yeast CLS by stimulating Rim15 **A.** Cells of the wild-type (WT) and indicated mutant strains were grown in the synthetic minimal YNB medium (0.67% Yeast Nitrogen Base without amino acids) initially containing 2% glucose, in the presence of 0.1% PE12 (ethanol was used as a vehicle at the final concentration of 0.5%) or in its absence (cells were subjected to ethanol-mock treatment). Survival curves of chronologically aging WT and mutant strains cultured with or without 0.1% PE12 are shown. Data are presented as means ± SEM (*n* = 8). The dotted line indicates the predicted survival curve of a particular mutant strain cultured with PE12 if this PE exhibits an additive longevity-extending effect with the mutation. Data for the mock-treated WT strain are replicated in all graphs of this Figure and in all graphs of Figure 8. Data for each of the mock-treated mutant strains presented in this Figure are replicated in the corresponding graphs of Figure 8. Data for the WT strain cultured with PE12 are replicated in all graphs of this Figure. **B.** The effect of PE12 on the signaling pathways and protein kinases integrated into the longevity-defining network. This effect is inferred from the data presented in **A.**, Tables 2 and 3, and Suppl. Figs. S5 and S11. Abbreviations: as in the legend to Figure 1.

**Figure 8:**
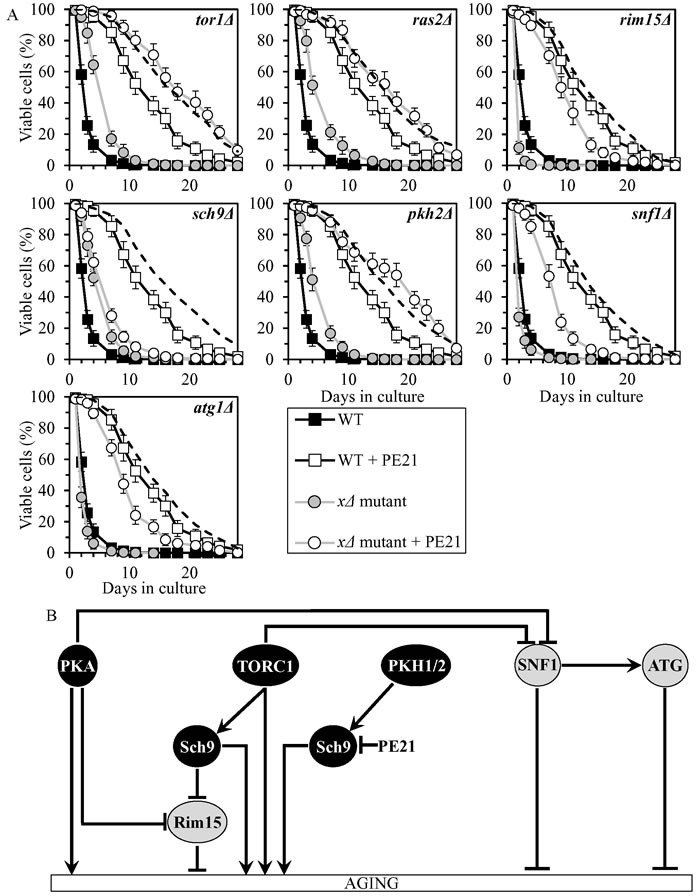
PE21 extends yeast CLS by attenuating a PKH1/2-sensitive form of Sch9 **A.** Cells of the wild-type (WT) and indicated mutant strains were grown in the synthetic minimal YNB medium (0.67% Yeast Nitrogen Base without amino acids) initially containing 2% glucose, in the presence of 0.1% PE21 (ethanol was used as a vehicle at the final concentration of 0.5%) or in its absence (cells were subjected to ethanol-mock treatment). Survival curves of chronologically aging WT and mutant strains cultured with or without 0.1% PE21 are shown. Data are presented as means ± SEM (*n* = 8). The dotted line indicates the predicted survival curve of a particular mutant strain cultured with PE21 if this PE exhibits an additive longevity-extending effect with the mutation. Data for the mock-treated WT strain are replicated in all graphs of this Figure and in all graphs of Figure 7. Data for each of the mock-treated mutant strains presented in this Figure are replicated in the corresponding graphs of Figure 7. Data for the WT strain cultured with PE21 are replicated in all graphs of this Figure. **B.** The effect of PE21 on the signaling pathways and protein kinases integrated into the longevity-defining network. This effect is inferred from the data presented in **A.**, Tables 2 and 3, and Suppl. Figs. S6 and S12. Abbreviations: as in the legend to Figure 1.

Survival curves shown in Figs. 3A–8A were compared. The survival curve of a strain cultured with the indicated PE was considered statistically different from the survival curve of the same strain cultured without it if the *p* value was less than 0.05. For each pair of survival curves with the *p* value less than 0.05, the survival rate of the strain cultured with the indicated PE was higher than the survival rate of the same strain cultured without it. The *p* values for comparing pairs of survival curves were calculated as described in Materials and methods.

**Table 3:** *p* Values for pairs of survival curves of the wild-type (WT) and mutant strain, both cultured in the presence of the indicated PE

**PE4**	*tor1Δ* + PE4	*ras2Δ* + PE4	*rim15Δ* + PE4	*sch9Δ* + PE4	*pkh2Δ* + PE4	*snf1Δ* + PE4	*atg1Δ* + PE4
WT + PE4	0.0827	**0.0002**	**< 0.0001**	**0.0004**	**0.0007**	**< 0.0001**	**< 0.0001**
**PE5**	*tor1Δ* + PE5	*ras2Δ* + PE5	*rim15Δ* + PE5	*sch9Δ* + PE5	*pkh2Δ* + PE5	*snf1Δ* + PE5	*atg1Δ* + PE5
WT + PE5	**< 0.0001**	0.0724	**< 0.0001**	**0.0008**	**< 0.0001**	**< 0.0001**	**0.0004**
**PE6**	*tor1Δ* + PE6	*ras2Δ* + PE6	*rim15Δ* + PE6	*sch9Δ* + PE6	*pkh2Δ* + PE6	*snf1Δ* + PE6	*atg1Δ* + PE6
WT + PE6	**< 0.0001**	**< 0.0001**	**0.0364**	**0.0008**	**< 0.0001**	**< 0.0001**	**< 0.0001**
**PE8**	*tor1Δ* + PE8	*ras2Δ* + PE8	*rim15Δ* + PE8	*sch9Δ* + PE8	*pkh2Δ* + PE8	*snf1Δ* + PE8	*atg1Δ* + PE8
WT + PE8	**< 0.0001**	**0.0124**	**< 0.0001**	**0.0032**	**< 0.0001**	**< 0.0001**	**0.0008**
**PE12**	*tor1Δ* + PE12	*ras2Δ* + PE12	*rim15Δ* + PE12	*sch9Δ* + PE12	*pkh2Δ* + PE12	*snf1Δ* + PE12	*atg1Δ* + PE12
WT + PE12	**0.0003**	**0.0011**	**< 0.0001**	**0.0018**	**< 0.0001**	**0.0004**	**0.0012**
**PE21**	*tor1Δ* + PE21	*ras2Δ* + PE21	*rim15Δ* + PE21	*sch9Δ* + PE21	*pkh2Δ* + PE21	*snf1Δ* + PE21	*atg1Δ* + PE21
WT + PE21	**0.0036**	**0.0062**	**0.0109**	**< 0.0001**	**0.0044**	**0.0009**	**0.0086**

Survival curves shown in Figs. 3A–8A were compared. The survival curve for the WT strain cultured with the indicated PE was considered statistically different from the survival curve for the mutant strain cultured with this PE if the *p* value was less than 0.05. The *p* values less than 0.05 are displayed in red color if the survival rate of the mutant strain cultured with the indicated PE was higher than the survival rate of the WT strain cultured with this PE. The *p* values less than 0.05 are displayed in blue color if the survival rate of the mutant strain cultured with the indicated PE was lower than the survival rate of the WT strain cultured with this PE. The *p* values for comparing pairs of survival curves were calculated as described in Materials and methods.

### PE5 slows chronological aging by impeding two branches of the PKA pathway

PE5 displayed an additive longevity-extending effect with the *sch9Δ* mutation, and increased yeast CLS in synergy with the *tor1Δ* and *pkh2Δ* mutations (Figure 4A, Tables 2 and 3, Suppl. Figure S2; note that data for the mock-treated WT strain and for the WT strain cultured with PE5 are replicated in all graphs of Figure 4A and Suppl. Figure S2). PE5 decreased the values of α and increased the values of MRDT for chronologically aging cultures of strains carrying each of these three mutations (Suppl. Figure S8; note that data for the mock-treated WT strain and for the WT strain cultured with PE5 are replicated in all graphs of this figure). Therefore, PE5 slows aging not through TORC1, PKH1/2 or Sch9.

PE5 increased CLS of the *rim15Δ*, *snf1Δ* and *atg1Δ* mutant strains, however to a lesser extent than that of WT strain (Figure 4A, Tables 2 and 3, Suppl. Figure S2). PE5 decreased the value of α and increased the value of MRDT strains carrying each of these mutations, although not as considerably as for WT (Suppl. Figure S8). Thus, PE5 slows aging independently of Rim15, SNF1 and ATG.

PE5 was unable to extend the CLS of *ras2Δ* (Figure 3A, Tables 2 and 3, Suppl. Figure S2) and did not alter the values of α or MRDT for this mutant strains (Suppl. Figure S8). Hence, PE5 delays aging by weakening two branches of the PKA signaling pathway (Figure 4B). One of these branches involves the Rim15-independent processes of autophagy inhibition and protein translation activation in the cytosol, whereas the other branch attenuates the Rim15-diven establishment of an anti-aging transcriptional program of numerous nuclear genes [8, 11, 34, 40, 61, 62, 66, 69, 72, 73, 84–86].

### PE6 delays chronological aging by coordinating processes that are not integrated into the network of longevity-defining signaling pathways/protein kinases

PE6 exhibited additive longevity-extending effects with the *rim15Δ*, *sch9Δ* and *atg1Δ* mutations, and extended longevity synergistically with the *tor1Δ*, *ras2Δ*, *pkh2Δ* and *snf1Δ* mutations (Figure 5A, Tables 2 and 3, Suppl. Figure S3; note that data for the mock-treated WT strain and for the WT strain cultured with PE6 are replicated in all graphs of Figure 5A and Suppl. Figure S3). PE6 lowered the values of α and raised the values of MRDT for chronologically aging cultures of strains carrying each of these seven mutations (Suppl. Figure S9; note that data for the mock-treated WT strain and for the WT strain cultured with PE6 are replicated in all graphs of this figure). Thus, PE6 delays aging by activating anti-aging processes and/or inhibiting pro-aging processes that are not assimilated into the network of presently known signaling pathways/protein kinases (Figure 5B).

Although *rim15Δ*, *snf1Δ* and *atg1Δ* exhibited decreased CLS in the absence of PE6, this PE extended the CLS of each of these mutant strains to a greater extent than that of WT strain (Figure 5A, Tables 2 and 3, Suppl. Figs. S3 and S9). It is possible that the efficiency with which PE6 activates anti-aging processes and/or inhibits pro-aging processes outside of the network in the absence of Rim15, Snf1 or Atg1 may exceed such efficiency in the presence of any of these anti-aging proteins.

### PE8 slows chronological aging by weakening the inhibitory effect of PKA on SNF1

PE8 displayed an additive longevity-extending effect with the *sch9Δ* mutation, and increased yeast CLS in synergy with the *tor1Δ* and *pkh2Δ* mutations (Figure 6A, Tables 2 and 3, Suppl. Figure S4; note that data for the mock-treated WT strain and for the WT strain cultured with PE8 are replicated in all graphs of Figure 6A and Suppl. Figure S4). PE8 decreased the values of α and increased the values of MRDT for chronologically aging cultures of strains carrying each of these three mutations (Suppl. Figure S10; note that data for the mock-treated WT strain and for the WT strain cultured with PE8 are replicated in all graphs of this figure). Therefore, PE8 slows aging independently of Sch9, TORC1 and PKH1/2.

PE8 increased CLS of the *rim15Δ* and *atg1Δ* mutant strains, though not as considerably as for WT (Figure 6A, Tables 2 and 3, Suppl. Figure S4). PE8 lowered the values of α and raised the values of MRDT for chronologically aging cultures of strains carrying each of these mutations (Suppl. Figure S10). Hence, PE8 delays aging not through Rim15 or ATG.

PE8 was unable to extend the CLS of *ras2Δ* and *snf1Δ* (Figure 6A, Tables 2 and 3, Suppl. Figure S4) and did not alter the values of α or MRDT for these mutant strains (Suppl. Figure S10). Thus, PE8 slows aging *via* PKA and SNF1, by attenuating the known inhibitory action of PKA on SNF1 (Figure 6B).

### PE12 delays chronological aging by stimulating Rim15

PE12 increased yeast CLS synergistically with the *pkh2Δ* mutation, and displayed additive longevity-extending effects with the *tor1Δ*, *ras2Δ* and *sch9Δ* mutations (Figure 7A, Tables 2 and 3, Suppl. Figure S5; note that data for the mock-treated WT strain and for the WT strain cultured with PE12 are replicated in all graphs of Figure 7A and Suppl. Figure S5). PE12 reduced the values of α and augmented the values of MRDT for chronologically aging cultures of strains carrying each of these four mutations (Suppl. Figure S11; note that data for the mock-treated WT strain and for the WT strain cultured with PE12 are replicated in all graphs of this figure). Hence, PE12 slows aging not through PKH1/2, TORC1, PKA or Sch9.

PE12 extended longevities of the *snf1Δ* and *atg1Δ* mutant strains, although to a lesser extent than that of WT strain (Figure 7A, Tables 2 and 3, Suppl. Figure S5). PE12 decreased the value of α and increased the value of MRDT strains carrying each of these mutations, however not as considerably as for WT (Suppl. Figure S11). Therefore, PE12 delays aging independently of SNF1 and ATG.

PE12 was unable to extend the CLS of *rim15Δ* (Figure 7A, Tables 2 and 3, Suppl. Figure S5) and did not alter the values of α or MRDT for this mutant strain (Suppl. Figure S11). Hence, PE12 slows aging by activating Rim15 (Figure 7B).

### PE21 slows chronological aging by inhibiting a PKH1/2-sensitive form of Sch9

PE21 increased yeast CLS in synergy with the *pkh2Δ* mutation, and displayed additive longevity-extending effects with the *tor1Δ* and *ras2Δ* mutations (Figure 8A, Tables 2 and 3, Suppl. Figure S6; note that data for the mock-treated WT strain and for the WT strain cultured with PE21 are replicated in all graphs of Figure 8A and Suppl. Figure S6). PE21 decreased the values of α and increased the values of MRDT for chronologically aging cultures of strains carrying each of these three mutations (Suppl. Figure S12; note that data for the mock-treated WT strain and for the WT strain cultured with PE21 are replicated in all graphs of this figure). Thus, PE21 slows aging independently of PKH1/2, TORC1 and PKA.

PE21 increased CLS of the *rim15Δ*, *snf1Δ* and *atg1Δ* mutant strains, although to a slightly lesser extent than that of WT (Figure 8A, Tables 2 and 3, Suppl. Figure S6). PE12 lowered the values of α and raised the values of MRDT for chronologically aging cultures of strains carrying each of these mutations, however somewhat less considerably than those for WT (Suppl. Figure S12). Hence, PE12 delays aging not through Rim15, SNF1 or ATG.

PE21 extended the CLS (Figure 8A, Tables 2 and 3, Suppl. Figure S6), decreased the value of α (Suppl. Figure S12) and increased the value of MRDT (Suppl. Figure S12) significantly less efficiently for *sch9Δ* than it did for WT. We therefore concluded that PE21 slows aging by attenuating a PKH1/2-sensitive form of Sch9 (Figure 8B).

## DISCUSSION

**Figure 9:**
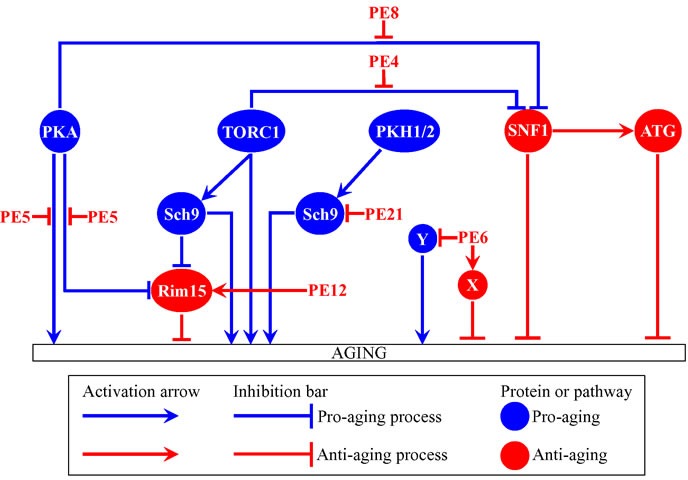
A model for how PE4, PE5, PE6, PE8, PE12 and PE21 delay yeast chronological aging *via* the longevity-defining network of signaling pathways/protein kinases Activation arrows and inhibition bars denote pro-aging processes (displayed in blue color) or anti-aging processes (displayed in red color). Pro-aging or anti-aging signaling pathways and protein kinases are displayed in blue or red color, respectively. Please see text for additional details. Abbreviations: as in the legend to Figure 1.

A hypothetical model for how the six PEs delay yeast chronological aging *via* the longevity-defining network of signaling pathways/protein kinases emerges from our analysis. This model is depicted schematically in Figure 9. The model suggests that these PEs delay aging as follows: 1) PE4 attenuates the inhibitory effect of TORC1 on SNF1; 2) PE5 weakens both the Rim15-dependent and Rim15-independent branches of the PKA signaling pathway; 3) PE6 activates anti-aging processes and/or inhibits pro-aging processes that are not integrated into the network of signaling pathways/protein kinases; 4) PE8 attenuates the inhibitory effect of PKA on SNF1; 5) PE12 activates Rim15; and 6) PE21 inhibits a PKH1/2-sensitive form of Sch9. Thus, geroprotective chemical compounds from some plants can slow yeast chronological aging by targeting different hubs, nodes and/or links of the longevity-defining network that integrates certain evolutionarily conserved signaling pathways and protein kinases. In the future, it would be important to validate the above hypothesis by investigating how each of the six aging-delaying PEs impacts the physical links that connect individual hubs and nodes of the chronological aging network shown in Figure 9. These links are known to be mainly activating or inhibiting phosphorylations and dephosphorylations of certain target proteins that are transiently or permanently reside in various cellular locations, including the plasma membrane, vacuole, nucleus, mitochondria or cytosol [1, 2, 5, 6, 10–12, 14, 21–76, 84–86].

Of note, we found that each of the six PEs delays aging through different signaling pathways and/or protein kinases (Figure 9). It is possible therefore that if these PEs are mixed in various combinations, some of the combinations may display additive or synergistic effects on the aging-delaying efficiencies of each other. Our ongoing studies explore this possibility.

This study also revealed that certain combinations of PE4, PE5, PE8, PE12 or PE21 and the *tor1Δ*, *ras2Δ*, *pkh2Δ* or *sch9Δ* mutation (each of which impairs a pro-aging signaling pathway or protein kinase) markedly increase aging-delaying proficiencies of each other. Furthermore, all combinations of PE6 and mutations impairing either anti-aging or pro-aging signaling pathways/protein kinases display additive or synergistic effects on the extent of aging delay. It is known that the network of longevity-defining signaling pathways/protein kinases is controlled by such aging-delaying chemical compounds as resveratrol, rapamycin, caffeine, spermidine, myriocin, methionine sulfoxide, lithocholic acid and cryptotanshinone [3, 5, 6, 8–10, 12, 14, 43, 54, 74–78]. One could envision therefore that certain combinations of these chemical compounds and the six PEs may have additive or synergistic effects on the aging-delaying proficiencies of each other. Our ongoing studies address the validity of this assumption.

The evolutionarily conserved nutrient-sensing signaling pathways that accelerate chronological aging in yeast (Figure 9) are known to stimulate chronological senescence and geroconversion of post-mitotic human cells; these pathways are likely to expedite organismal aging and cancer development in humans [87–93]. Moreover, genetic and pharmacological manipulations that attenuate these signaling pathways and delay chronological aging in yeast are known to decelerate chronological senescence and geroconversion of post-mitotic human cells; it is believed that these manipulations may also delay organismal aging and tumorigenesis in humans [87–93]. Thus, some of the six geroprotective PEs that slow down yeast chronological aging through these signaling pathways (Figure 9) may prolong healthy lifespan and decelerate tumorigenesis.

The challenge for the future is to assess whether any of the six PEs can delay the onset and progression of chronic diseases associated with human aging. Among such diseases are arthritis, diabetes, heart disease, kidney disease, liver dysfunction, sarcopenia, stroke, neurodegenerative diseases (including Parkinson's, Alzheimer's and Huntington's diseases), and many forms of cancer [3, 6, 9, 12, 15–18, 21, 79, 94–105]. Because the major aspects of aging and age-related pathology are conserved across phyla [1, 4–6, 10–13, 16–18], it is noteworthy that this study, recent findings [78] and our ongoing research have revealed several features of the six PEs as potential interventions for decelerating chronic diseases of old age. These features are the following: 1) the six PEs are caloric restriction (CR) mimetics that imitate the aging-delaying effects of the CR diet in yeast under non-CR conditions; 2) they are geroprotectors that slow yeast aging by eliciting a hormetic stress response; 3) they extend yeast longevity more efficiently than any lifespan-prolonging chemical compound yet described; 4) they delay aging through signaling pathways and protein kinases implicated in such age-related pathologies as type 2 diabetes, neurodegenerative diseases, cardiac hypertrophy, cardiovascular disease, sarcopenia and cancers; and 5) they extend longevity and delay the onset of age-related diseases in other eukaryotic model organisms. The potential of using the six aging-delaying PEs for delaying the onset of age-related diseases in humans is further underscored by the fact that the Health Canada government agency classifies these PEs as safe for human consumption and recommends to use five of them as health-improving supplements with clinically proven benefits to human health [106].

## MATERIALS AND METHODS

### Yeast strains, media and growth conditions

The wild-type strain *Saccharomyces cerevisiae* BY4742 (*MAT*α *his3*Δ*1 leu2*Δ*0 lys2*Δ*0 ura3*Δ*0*) and single-gene-deletion mutant strains in the BY4742 genetic background (all from Thermo Scientific/Open Biosystems) were grown in a synthetic minimal YNB medium (0.67% (w/v) Yeast Nitrogen Base without amino acids) initially containing 2% (w/v) glucose and supplemented with 20 mg/l histidine, 30 mg/l leucine, 30 mg/l lysine and 20 mg/l uracil. Cells were cultured at 30°C with rotational shaking at 200 rpm in Erlenmeyer flasks at a “flask volume/medium volume” ratio of 5:1.

### Aging-delaying PEs

0.5% (w/v) PE4 from *Cimicifuga racemosa*, 0.5% (w/v) PE5 from *Valeriana officinalis L.*, 1.0% (w/v) PE6 from *Passiflora incarnata L.*, 0.3% (w/v) PE8 from *Ginkgo biloba*, 0.1% (w/v) PE12 from *Apium graveolens L.* and 0.1% (w/v) PE21 from *Salix alba* were used [78]. A 20% (w/v) stock solution of each PE in ethanol was made on the day of adding this PE to cell cultures. For each PE, the stock solution was added to growth medium with 2% (w/v) glucose immediately following cell inoculation into the medium.

### CLS assay

A sample of cells was taken from a culture at a certain day following cell inoculation and PE addition into the medium. A fraction of the sample was diluted in order to determine the total number of cells using a hemacytometer. Another fraction of the cell sample was diluted and serial dilutions of cells were plated in duplicate onto YEP (1% (w/v) yeast extract, 2% (w/v) peptone) plates containing 2% (w/v) glucose as carbon source. After 2 d of incubation at 30°C, the number of colony forming units (CFU) per plate was counted. The number of CFU was defined as the number of viable cells in a sample. For each culture, the percentage of viable cells was calculated as follows: (number of viable cells per ml/total number of cells per ml) × 100. The percentage of viable cells in mid-logarithmic growth phase was set at 100%.

### Miscellaneous procedures

The age-specific mortality rate (q_x_) [79, 81], Gompertz slope or mortality rate coefficient (α) [80, 81], and mortality rate doubling time (MRDT) [80, 81] were calculated as previously described. The value of q_x_ was calculated as the number of cells that lost viability (i.e. are unable to form a colony on the surface of a solid nutrient-rich medium) during each time interval divided by the number of viable (i.e. clonogenic) cells at the end of the interval. The natural logarithms of the values of q_x_ for each time interval were plotted against time. The value of α was calculated as the slope of the Gompertz mortality line, whereas the value of MRDT was calculated as ln2/α.

### Statistical analysis

Statistical analysis was performed using Microsoft Excel's (2010) Analysis ToolPack-VBA. All data on cell survival are presented as mean ± SEM. The *p* values for comparing the means of two groups (using an unpaired two-tailed *t* test) and survival curves (using a two-tailed *t* test) were calculated with the help of the GraphPad Prism statistics software.

## SUPPLEMENTARY MATERIAL AND FIGURES


