# Supplementary material for: Six plant extracts delay yeast chronological aging through different signaling pathways
Source: Oncotarget. 2016 Jul 18;7(32):50845–63. doi: 10.18632/oncotarget.10689 (PMC5239441; doi:10.18632/oncotarget.10689)
Supplement: Supplementary file 1 [file oncotarget-07-50845-s001.pdf]

Six plant extracts delay yeast chronological aging through different signaling pathways

Supplementary Material

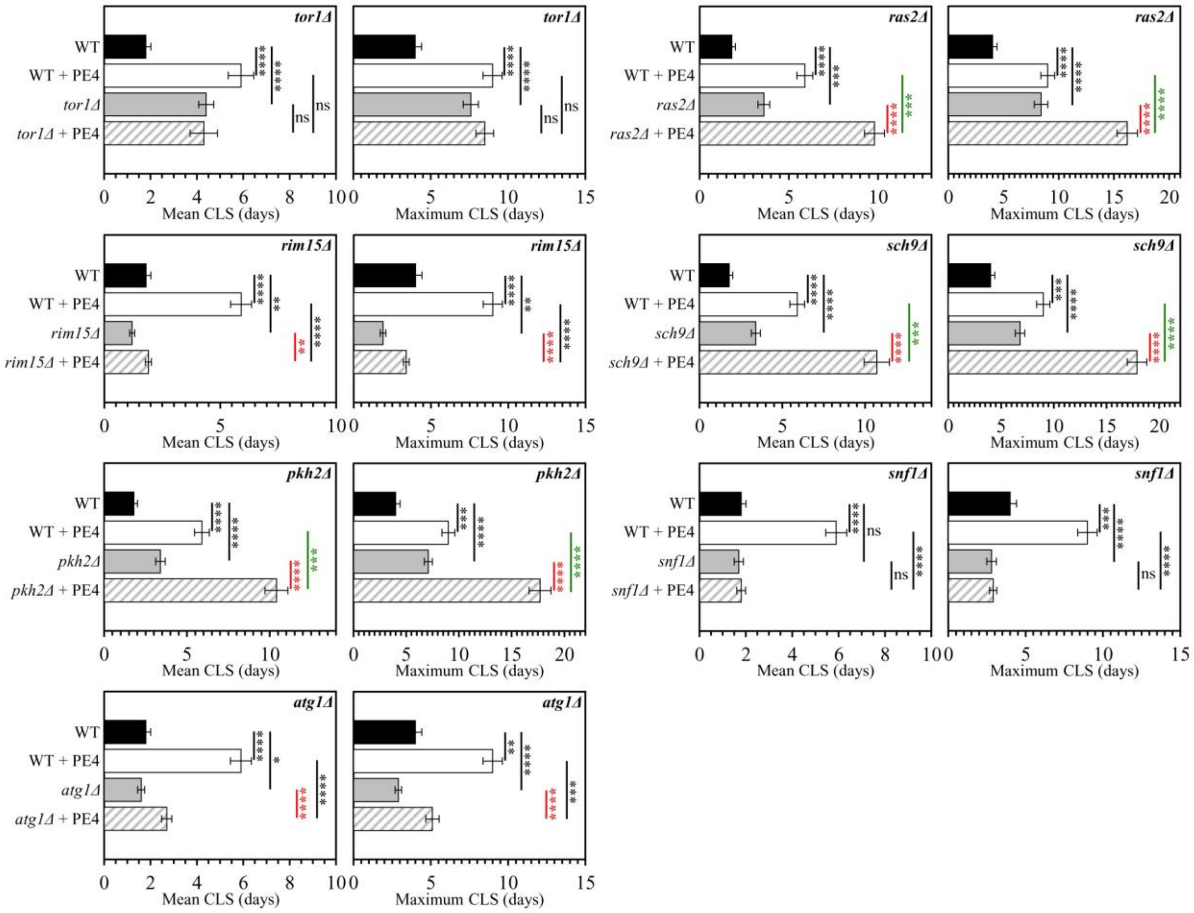

Supplementary Figure S1. PE4 is unable to extend the chronological lifespans (CLS) of the *tor1Δ* and *snf1Δ* mutant strains, and exhibits additive CLS-extending effects with the *ras2Δ, sch9Δ* and *pkh2Δ* mutations. Cells of the wild-type (WT) and indicated mutant strains were grown in the synthetic minimal YNB medium (0.67% Yeast Nitrogen Base without amino acids) initially containing 2% glucose, in the presence of 0.5% PE4 (ethanol was used as a vehicle at the final concentration of 2.5%) or in its absence (cells were subjected to ethanol-mock treatment). Survival curves shown in Fig. 3A were used to calculate the mean and maximum CLS for WT and mutant strains cultured with or without 0.5% PE4. Data are presented as means ± SEM (n = 7; ns, not significant; *$p < 0.05$; **p < 0.01; ***$p <$ 0.001; ****$p < 0.0001$). The ability of PE4 to cause a significant (*$p < 0.05$; **p < 0.01; ***$p < 0.001$; ****$p < 0.0001$) increase in the CLS of a particular mutant strain is displayed in red color. The ability of a combination between PE4 and a particular mutation to cause a significant (*$p < 0.05$; **p < 0.01; ***$p < 0.001$; ****$p < 0.0001$) increase in CLS-extending efficiencies of each other (i.e. the ability of such combination to exhibit an additive extending effect on yeast CLS) is displayed in green color. Data for the mock-treated WT strain are replicated in all graphs of this Supplementary Figure and in all graphs of Supplementary Figure S2. Data for each of the mock-treated mutant strains presented in this Supplementary Figure are replicated in the corresponding graphs of Supplementary Figure S2. Data for the WT strain cultured with PE4 are replicated in all graphs of this Supplementary Figure.

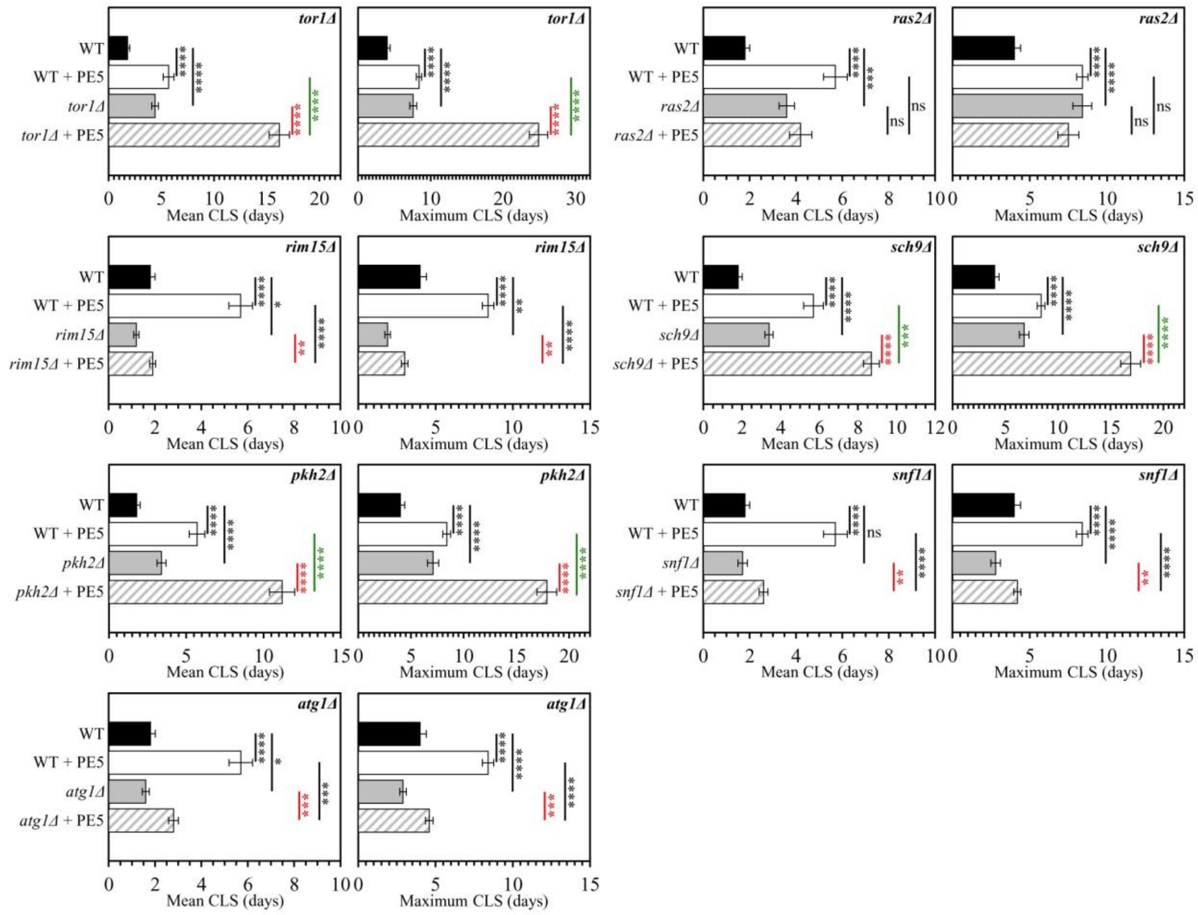

Supplementary Figure S2. PE5 is unable to extend the chronological lifespan (CLS) of the *ras2Δ* mutant strain, exhibits an additive CLS-extending effect with the *sch9Δ* mutation, and increases yeast CLS in synergy with the *tor1Δ* and *pkh2Δ* mutations. Cells of the wild-type (WT) and indicated mutant strains were grown in the synthetic minimal YNB medium (0.67% Yeast Nitrogen Base without amino acids) initially containing 2% glucose, in the presence of 0.5% PE5 (ethanol was used as a vehicle at the final concentration of 2.5%) or in its absence (cells were subjected to ethanol-mock treatment). Survival curves shown in Fig. 4A were used to calculate the mean and maximum CLS for WT and mutant strains cultured with or without 0.5% PE5. Data are presented as means ± SEM (n = 7; ns, not significant; *$p < 0.05$; **p < 0.01; ***$p < 0.001$; ****$p < 0.0001$). The ability of PE5 to cause a significant (*$p < 0.05$; **p < 0.01; ***$p < 0.001$; ****$p < 0.0001$) increase in the CLS of a particular mutant strain is displayed in red color. The ability of a combination between PE5 and a particular mutation to cause a significant (*$p < 0.05$; **p < 0.01; ***$p < 0.001$; ****$p < 0.0001$) increase in CLS-extending efficiencies of each other (i.e. the ability of such combination to exhibit an additive or synergistic CLS-extending effect) is displayed in green color. Data for the mock-treated WT strain are replicated in all graphs of this Supplementary Figure and in all graphs of Supplementary Figure S1. Data for each of the mock-treated mutant strains presented in this Supplementary Figure are replicated in the corresponding graphs of Supplementary Figure S1. Data for the WT strain cultured with PE5 are replicated in all graphs of this Supplementary Figure.

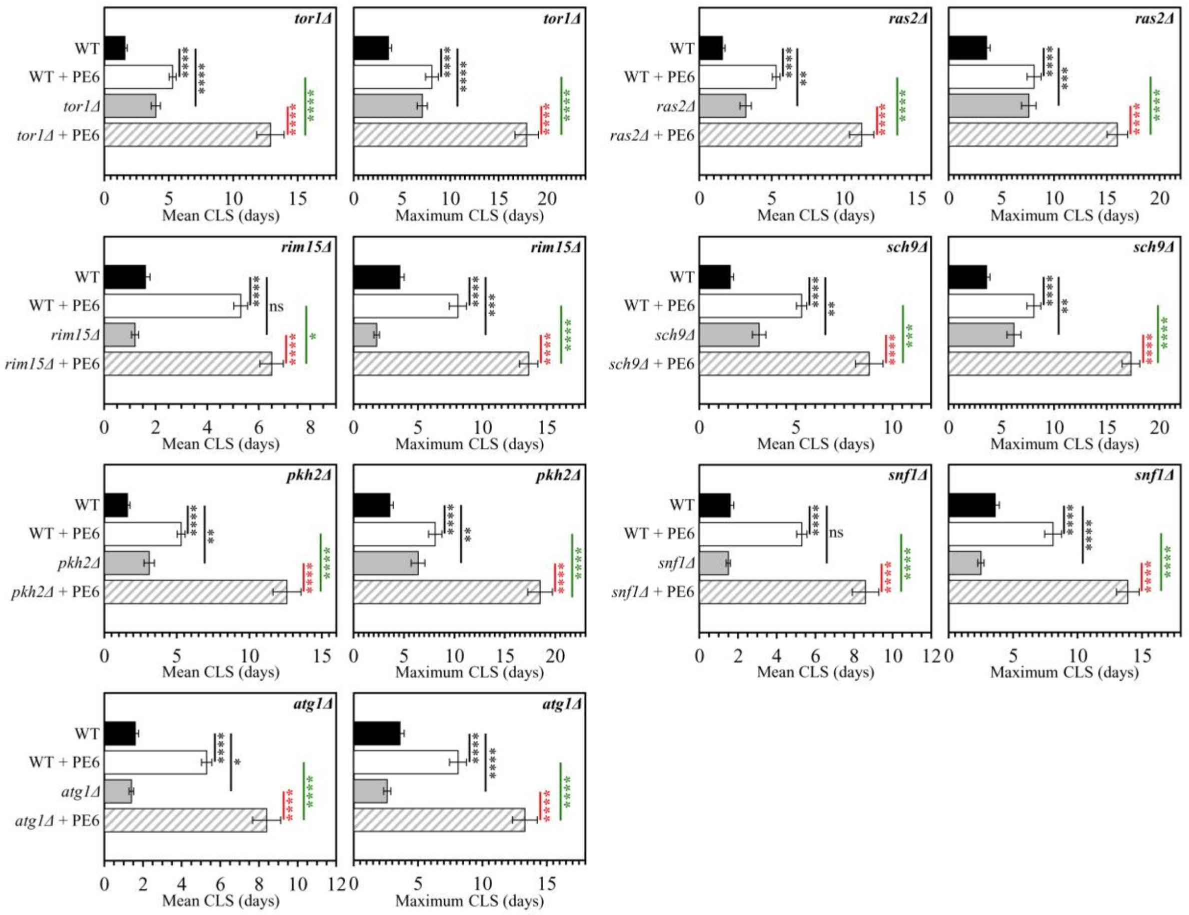

Supplementary Figure S3. PE6 exhibits additive CLS-extending effects with the *rim15Δ*, *sch9Δ* and *atg1Δ* mutations; PE6 also increases yeast CLS in synergy with the *tor1Δ*, *ras2Δ*, *pkh2Δ* and *snf1Δ* mutations. Cells of the wild-type (WT) and indicated mutant strains were grown in the synthetic minimal YNB medium (0.67% Yeast Nitrogen Base without amino acids) initially containing 2% glucose, in the presence of 1.0% PE6 (ethanol was used as a vehicle at the final concentration of 5.0%) or in its absence (cells were subjected to ethanol-mock treatment). Survival curves shown in Fig. 5A were used to calculate the mean and maximum CLS for WT and mutant strains cultured with or without 1.0% PE6. Data are presented as means ± SEM (n = 8; ns, not significant; **p* < 0.05; **p < 0.01; ****p* < 0.001; *****p* < 0.0001). The ability of PE6 to cause a significant (**p* < 0.05; **p < 0.01; ****p* < 0.001; *****p* < 0.0001) increase in the CLS of a particular mutant strain is displayed in red color. The ability of a combination between PE6 and a particular mutation to cause a significant (**p* < 0.05; **p < 0.01; ****p* < 0.001; *****p* < 0.0001) increase in CLS-extending efficiencies of each other (i.e. the ability of such combination to exhibit an additive or synergistic CLS-extending effect) is displayed in green color. Data for the mock-treated WT strain and for the WT strain cultured with PE6 are replicated in all graphs of this Supplementary Figure.

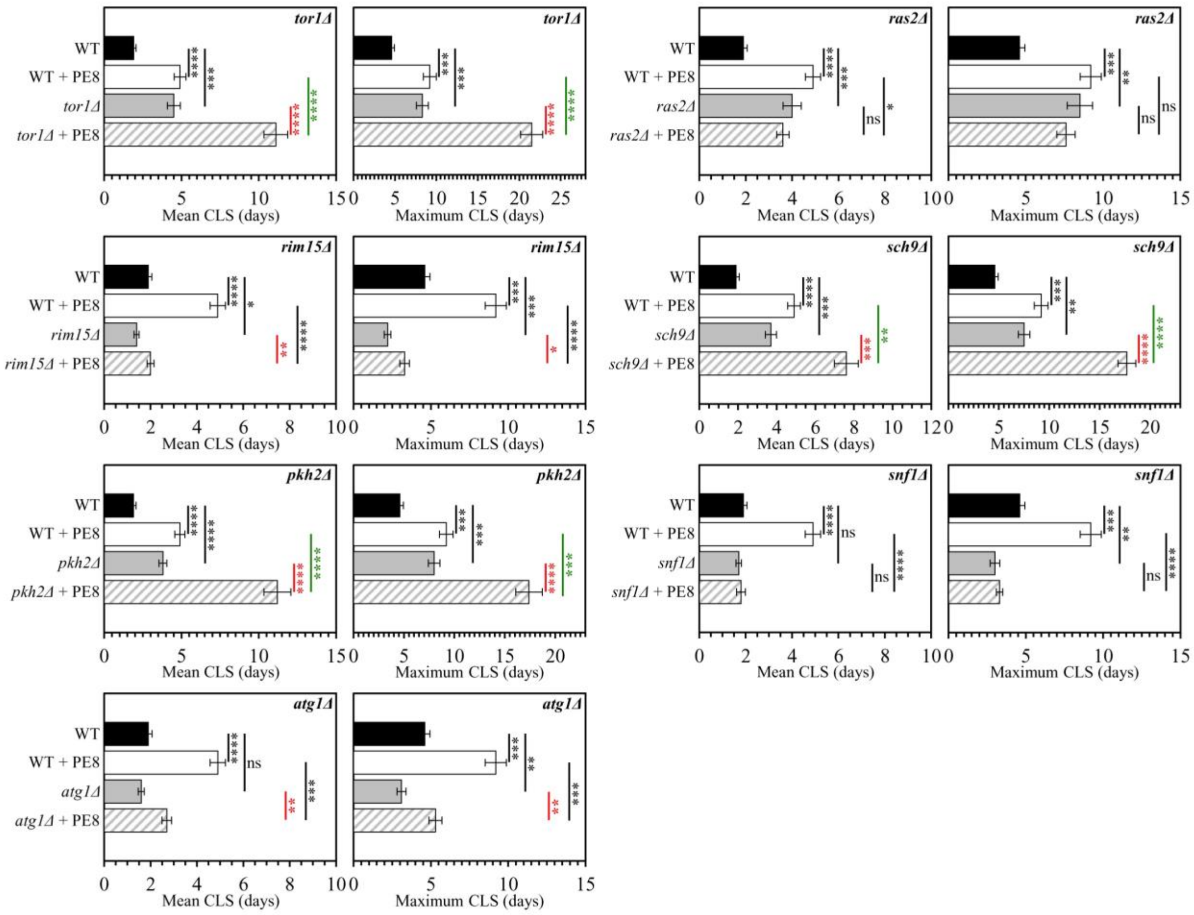

Supplementary Figure S4. PE8 is unable to extend the chronological lifespans (CLS) of the *ras2Δ* and *snf1Δ* mutant strains, exhibits an additive CLS-extending effect with the *sch9Δ* mutation, and increases yeast CLS in synergy with the *tor1Δ* and *pkh2Δ* mutations. Cells of the wild-type (WT) and indicated mutant strains were grown in the synthetic minimal YNB medium (0.67% Yeast Nitrogen Base without amino acids) initially containing 2% glucose, in the presence of 0.3% PE8 (ethanol was used as a vehicle at the final concentration of 1.5%) or in its absence (cells were subjected to ethanol-mock treatment). Survival curves shown in Fig. 6A were used to calculate the mean and maximum CLS for WT and mutant strains cultured with or without 0.3% PE8. Data are presented as means ± SEM (n = 6; ns, not significant; **p* < 0.05; **p < 0.01; ****p* < 0.001; *****p* < 0.0001). The ability of PE8 to cause a significant (**p* < 0.05; **p < 0.01; ****p* < 0.001; *****p* < 0.0001) increase in the CLS of a particular mutant strain is displayed in red color. The ability of a combination between PE8 and a particular mutation to cause a significant (**p* < 0.05; **p < 0.01; ****p* < 0.001; *****p* < 0.0001) increase in CLS-extending efficiencies of each other (i.e. the ability of such combination to exhibit an additive or synergistic CLS-extending effect) is displayed in green color. Data for the mock-treated WT strain and for the WT strain cultured with PE8 are replicated in all graphs of this Supplementary Figure.

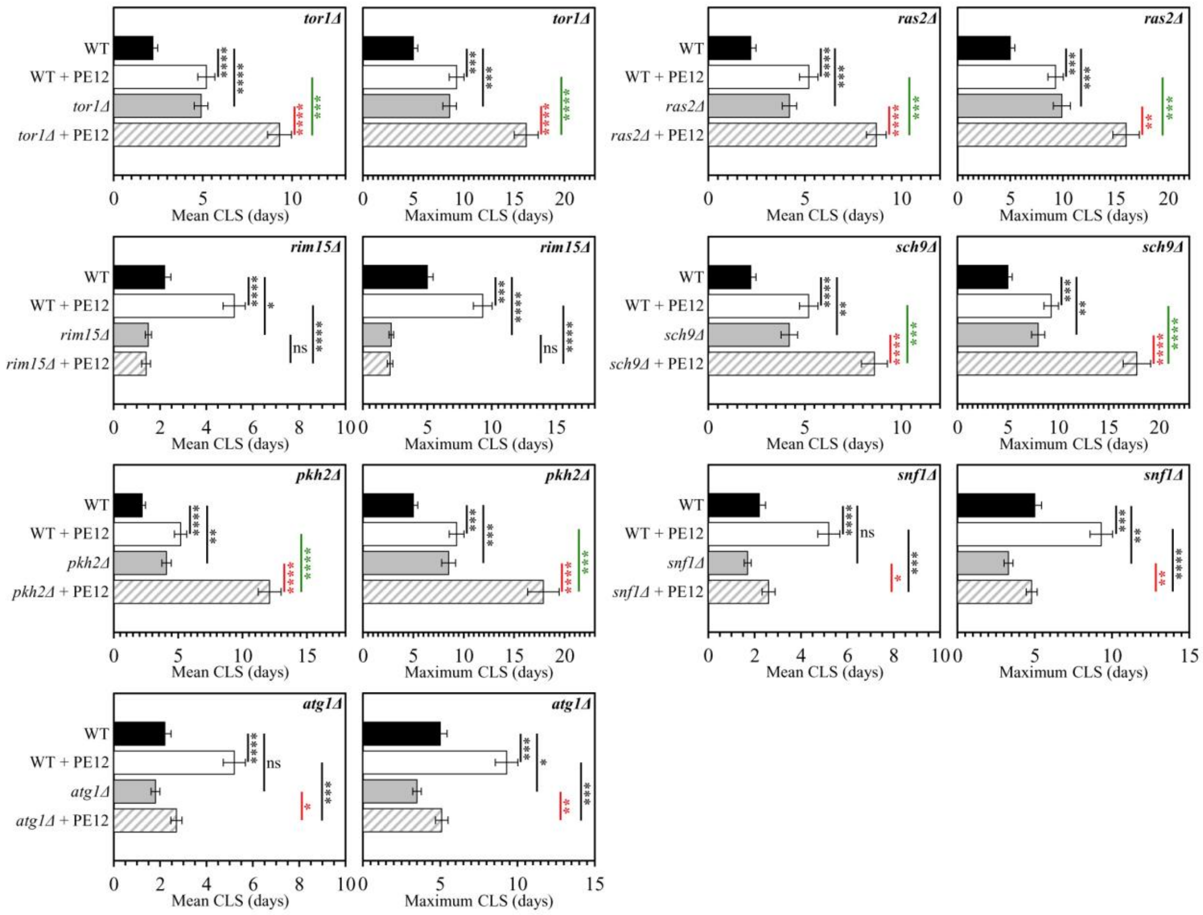

Supplementary Figure S5. PE12 is unable to extend the chronological lifespan (CLS) of the *rim15Δ* mutant strain, exhibits additive CLS-extending effects with the *tor1Δ*, *ras2Δ* and *sch9Δ* mutations, and increases yeast CLS in synergy with the *pkh2Δ* mutation. Cells of the wild-type (WT) and indicated mutant strains were grown in the synthetic minimal YNB medium (0.67% Yeast Nitrogen Base without amino acids) initially containing 2% glucose, in the presence of 0.1% PE12 (ethanol was used as a vehicle at the final concentration of 0.5%) or in its absence (cells were subjected to ethanol-mock treatment). Survival curves shown in Fig. 7A were used to calculate the mean and maximum CLS for WT and mutant strains cultured with or without 0.1% PE12. Data are presented as means ± SEM (n = 8; ns, not significant; *$p < 0.05$; **p < 0.01; ***$p < 0.001$; ****$p < 0.0001$). The ability of PE12 to cause a significant (*$p < 0.05$; **p < 0.01; ***$p < 0.001$; ****$p < 0.0001$) increase in the CLS of a particular mutant strain is displayed in red color. The ability of a combination between PE12 and a particular mutation to cause a significant (*$p < 0.05$; **p < 0.01; ***$p < 0.001$; ****$p < 0.0001$) increase in CLS-extending efficiencies of each other (i.e. the ability of such combination to exhibit an additive or synergistic CLS-extending effect) is displayed in green color. Data for the mock-treated WT strain are replicated in all graphs of this Supplementary Figure and in all graphs of Supplementary Figure S6. Data for each of the mock-treated mutant strains presented in this Supplementary Figure are replicated in the corresponding graphs of Supplementary Figure S6. Data for the WT strain cultured with PE12 are replicated in all graphs of this Supplementary Figure.

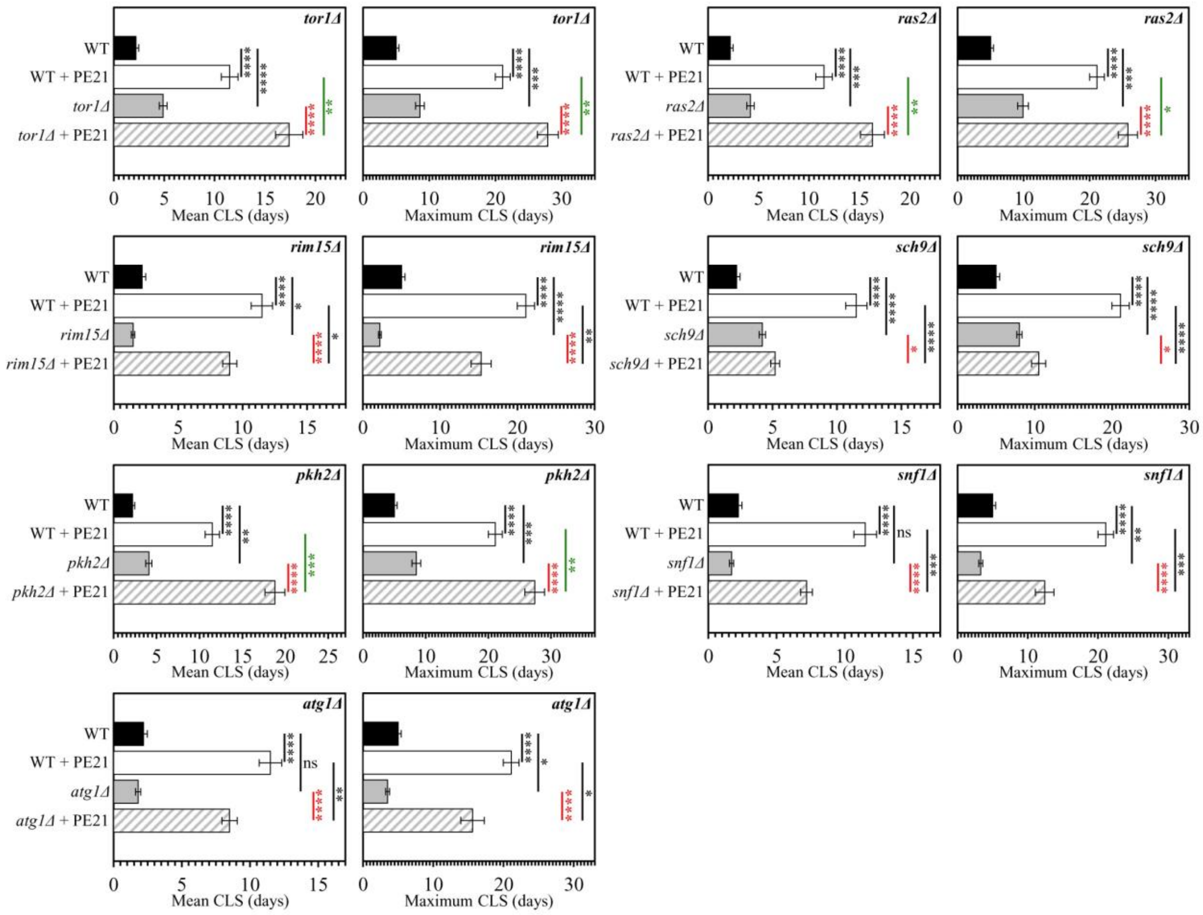

Supplementary Figure S6. PE21 extends the chronological lifespan (CLS) of the *sch9Δ* mutant strain significantly less efficiently than that of the wild-type (WT) strain, exhibits additive CLS-extending effects with the *tor1Δ* and *ras2Δ* mutations, and increases yeast CLS in synergy with the *pkh2Δ* mutation. Cells of the WT and indicated mutant strains were grown in the synthetic minimal YNB medium (0.67% Yeast Nitrogen Base without amino acids) initially containing 2% glucose, in the presence of 0.1% PE21 (ethanol was used as a vehicle at the final concentration of 0.5%) or in its absence (cells were subjected to ethanol-mock treatment). Survival curves shown in Fig. 8A were used to calculate the mean and maximum CLS for WT and mutant strains cultured with or without 0.1% PE21. Data are presented as means ± SEM (n = 42 for WT; n = 5-7 for WT with PE21 and mutants strains with or without PE; ns, not significant; *$p < 0.05$; **p < 0.01; ***$p < 0.001$; ****$p < 0.0001$). The ability of PE21 to cause a significant (*$p < 0.05$; **p < 0.01; ***$p < 0.001$; ****$p < 0.0001$) increase in the CLS of a particular mutant strain is displayed in red color. The ability of a combination between PE21 and a particular mutation to cause a significant (*$p < 0.05$; **p < 0.01; ***$p < 0.001$; ****$p < 0.0001$) increase in CLS-extending efficiencies of each other (i.e. the ability of such combination to exhibit an additive or synergistic CLS-extending effect) is displayed in green color. Data for the mock-treated WT strain are replicated in all graphs of this Supplementary Figure and in all graphs of Supplementary Figure S5. Data for each of the mock-treated mutant strains presented in this Supplementary Figure are replicated in the corresponding graphs of Supplementary Figure S5. Data for the WT strain cultured with PE21 are replicated in all graphs of this Supplementary Figure.

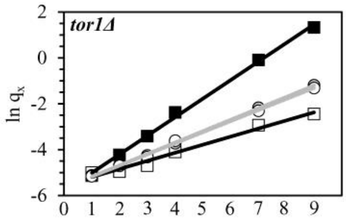

	α	MR DT	Rate of aging
WT	0.81	0.86	
WT + PE4	0.35	1.98	⇩ vs. WT
tor1Δ	0.50	1.39	⇩ vs. WT
tor1Δ + PE4	0.48	1.44	⇩ vs. WT<br>⬆ vs. WT + PE4<br>⇔ vs. *tor1Δ*

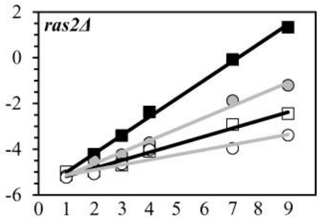

	α	MR DT	Rate of aging
WT	0.81	0.86	
WT + PE4	0.35	1.98	⇩ vs. WT
ras2Δ	0.51	1.36	⇩ vs. WT
ras2Δ + PE4	0.22	3.15	⇩ vs. WT<br>⇩ vs. WT + PE4<br>⇩ vs. *ras2Δ*

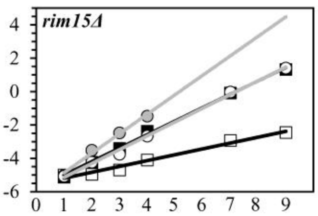

	α	MR DT	Rate of aging
WT	0.81	0.86	
WT + PE4	0.35	1.98	⇩ vs. WT
rim15Δ	1.17	0.60	⬆ vs. WT
rim15Δ + PE4	0.83	0.83	⇔ vs. WT<br>⬆ vs. WT + PE4<br>⇩ vs. *rim15Δ*

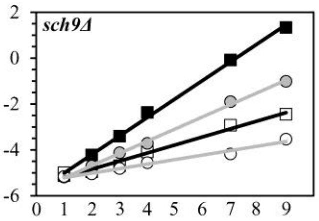

	α	MR DT	Rate of aging
WT	0.81	0.86	
WT + PE4	0.35	1.98	⇩ vs. WT
sch9Δ	0.53	1.31	⇩ vs. WT
sch9Δ + PE4	0.20	3.47	⇩ vs. WT<br>⇩ vs. WT + PE4<br>⇩ vs. *sch9Δ*

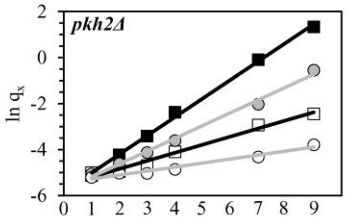

	α	MR DT	Rate of aging
WT	0.81	0.86	
WT + PE4	0.35	1.98	⇩ vs. WT
pkh2Δ	0.56	1.24	⇩ vs. WT
pkh2Δ + PE4	0.17	4.08	⇩ vs. WT<br>⇩ vs. WT + PE4<br>⇩ vs. *pkh2Δ*

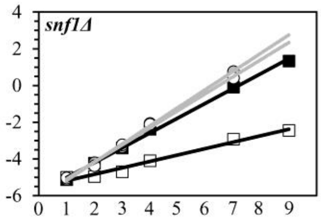

	α	MR DT	Rate of aging
WT	0.81	0.86	
WT + PE4	0.35	1.98	⇩ vs. WT
snf1Δ	0.99	0.70	⬆ vs. WT
snf1Δ + PE4	0.93	0.75	⬆ vs. WT<br>⬆ vs. WT + PE4<br>⇔ vs. *snf1Δ*

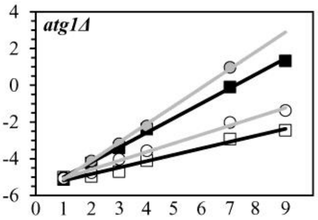

	α	MR DT	Rate of aging
WT	0.81	0.86	
WT + PE4	0.35	1.98	⇩ vs. WT
atg1Δ	1.00	0.69	⬆ vs. WT
atg1Δ + PE4	0.48	1.44	⇩ vs. WT<br>⬆ vs. WT + PE4<br>⇩ vs. *atg1Δ*

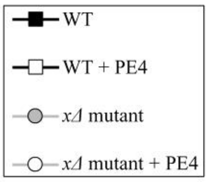

Supplementary Figure S7. Analysis of the Gompertz mortality function indicates that PE4 delays yeast chronological aging by attenuating the inhibitory effect of TORC1 on SNF1. Cells of the wild-type (WT) and indicated mutant strains were grown in the synthetic minimal YNB medium (0.67% Yeast Nitrogen Base without amino acids) initially containing 2% glucose, in the presence of 0.5% PE4 (ethanol was used as a vehicle at the final concentration of 2.5%) or in its absence (cells were subjected to ethanol-mock treatment). Survival curves shown in Fig. 3A were used to calculate the age-specific mortality rates (q_x), the Gompertz mortality rates (also known as mortality rate coefficient α) and the mortality rate doubling times (MRDT) for WT and mutant yeast populations cultured with or without 0.5% PE4. The values of q_x, α and MRDT were calculated as described in Materials and methods. Data for the mock-treated WT strain are replicated in all graphs of this Supplementary Figure and in all graphs of Supplementary Figure S8. Data for each of the mock-treated mutant strains presented in this Supplementary Figure are replicated in the corresponding graphs of Supplementary Figure S8. Data for the WT strain cultured with PE4 are replicated in all graphs of this Supplementary Figure.

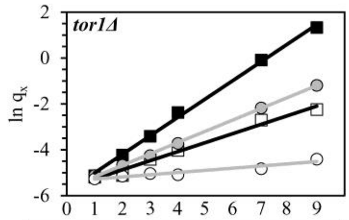

	α	MR DT	Rate of aging
WT	0.81	0.86	
WT + PE5	0.40	1.73	⇩ vs. WT
tor1Δ	0.50	1.39	⇩ vs. WT
tor1Δ + PE5	0.10	6.93	⇩ vs. WT<br>⇩ vs. WT + PE5<br>⇩ vs. *tor1Δ*

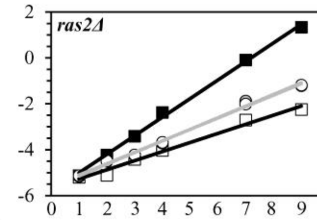

	α	MR DT	Rate of aging
WT	0.81	0.86	
WT + PE5	0.40	1.73	⇩ vs. WT
ras2Δ	0.51	1.36	⇩ vs. WT
ras2Δ + PE5	0.50	1.39	⇩ vs. WT<br>⬆ vs. WT + PE5<br>⇔ vs. *ras2Δ*

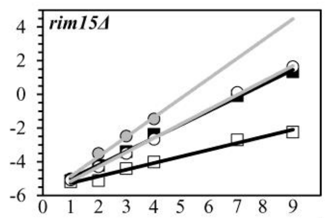

	α	MR DT	Rate of aging
WT	0.81	0.86	
WT + PE5	0.40	1.73	⇩ vs. WT
rim15Δ	1.17	0.60	⬆ vs. WT
rim15Δ + PE5	0.86	0.81	⬆ vs. WT<br>⬆ vs. WT + PE5<br>⇩ vs. *rim15Δ*

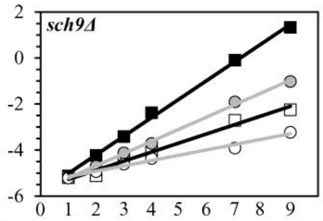

	α	MR DT	Rate of aging
WT	0.81	0.86	
WT + PE5	0.40	1.73	⇩ vs. WT
sch9Δ	0.53	1.31	⇩ vs. WT
sch9Δ + PE5	0.23	3.01	⇩ vs. WT<br>⇩ vs. WT + PE5<br>⇩ vs. *sch9Δ*

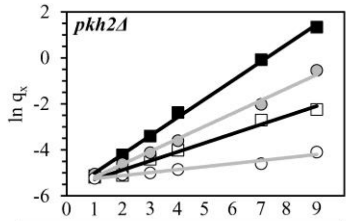

	α	MR DT	Rate of aging
WT	0.81	0.86	
WT + PE5	0.40	1.73	⇩ vs. WT
pkh2Δ	0.56	1.24	⇩ vs. WT
pkh2Δ + PE5	0.13	5.33	⇩ vs. WT<br>⇩ vs. WT + PE5<br>⇩ vs. *pkh2Δ*

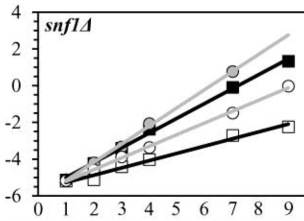

	α	MR DT	Rate of aging
WT	0.81	0.86	
WT + PE5	0.40	1.73	⇩ vs. WT
snf1Δ	0.99	0.70	⬆ vs. WT
snf1Δ + PE5	0.64	1.08	⇩ vs. WT<br>⬆ vs. WT + PE5<br>⇩ vs. *snf1Δ*

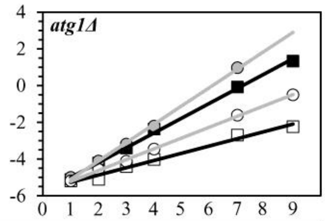

	α	MR DT	Rate of aging
WT	0.81	0.86	
WT + PE5	0.40	1.73	⇩ vs. WT
atg1Δ	1.00	0.69	⬆ vs. WT
atg1Δ + PE5	0.59	1.17	⇩ vs. WT<br>⬆ vs. WT + PE5<br>⇩ vs. *atg1Δ*

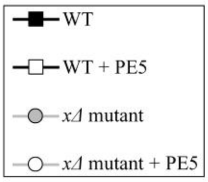

Supplementary Figure S8. Analysis of the Gompertz mortality function indicates that PE5 delays yeast chronological aging by attenuating two branches of the PKA signaling pathway. Cells of the wild-type (WT) and indicated mutant strains were grown in the synthetic minimal YNB medium (0.67% Yeast Nitrogen Base without amino acids) initially containing 2% glucose, in the presence of 0.5% PE5 (ethanol was used as a vehicle at the final concentration of 2.5%) or in its absence (cells were subjected to ethanol-mock treatment). Survival curves shown in Fig. 4A were used to calculate the age-specific mortality rates (q_x), the Gompertz mortality rates (also known as mortality rate coefficient α) and the mortality rate doubling times (MRDT) for WT and mutant yeast populations cultured with or without 0.5% PE5. The values of q_x, α and MRDT were calculated as described in Materials and methods. Data for the mock-treated WT strain are replicated in all graphs of this Supplementary Figure and in all graphs of Supplementary Figure S7. Data for each of the mock-treated mutant strains presented in this Supplementary Figure are replicated in the corresponding graphs of Supplementary Figure S7. Data for the WT strain cultured with PE5 are replicated in all graphs of this Supplementary Figure.

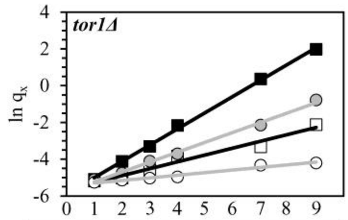

	α	MR DT	Rate of aging
WT	0.89	0.78	
WT + PE6	0.37	1.87	⇩ vs. WT
tor1Δ	0.54	1.28	⇩ vs. WT
tor1Δ + PE6	0.14	4.95	⇩ vs. WT<br>⇩ vs. WT + PE6<br>⇩ vs. *tor1Δ*

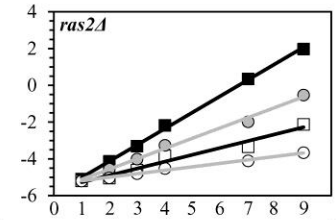

	α	MR DT	Rate of aging
WT	0.89	0.78	
WT + PE6	0.37	1.87	⇩ vs. WT
ras2Δ	0.56	1.24	⇩ vs. WT
ras2Δ + PE6	0.19	3.65	⇩ vs. WT<br>⇩ vs. WT + PE6<br>⇩ vs. *ras2Δ*

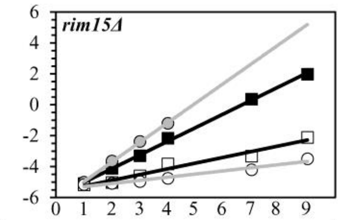

	α	MR DT	Rate of aging
WT	0.89	0.78	
WT + PE6	0.37	1.87	⇩ vs. WT
rim15Δ	1.27	0.55	⬆ vs. WT
rim15Δ + PE6	0.21	3.30	⇩ vs. WT<br>⇩ vs. WT + PE6<br>⇩ vs. *rim15Δ*

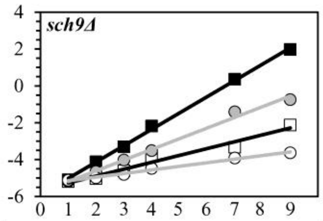

	α	MR DT	Rate of aging
WT	0.89	0.78	
WT + PE6	0.37	1.87	⇩ vs. WT
sch9Δ	0.58	1.19	⇩ vs. WT
sch9Δ + PE6	0.20	3.47	⇩ vs. WT<br>⇩ vs. WT + PE6<br>⇩ vs. *sch9Δ*

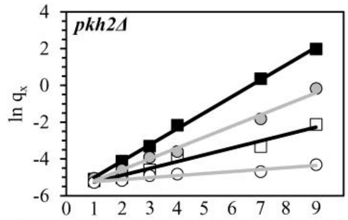

	α	MR DT	Rate of aging
WT	0.89	0.78	
WT + PE6	0.37	1.87	⇩ vs. WT
pkh2Δ	0.60	1.16	⇩ vs. WT
pkh2Δ + PE6	0.11	6.3	⇩ vs. WT<br>⇩ vs. WT + PE6<br>⇩ vs. *pkh2Δ*

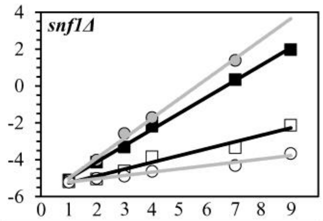

	α	MR DT	Rate of aging
WT	0.89	0.78	
WT + PE6	0.37	1.87	⇩ vs. WT
snf1Δ	1.08	0.64	⬆ vs. WT
snf1Δ + PE6	0.18	3.85	⇩ vs. WT<br>⇩ vs. WT + PE6<br>⇩ vs. *snf1Δ*

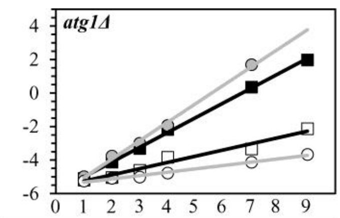

	α	MR DT	Rate of aging
WT	0.89	0.78	
WT + PE6	0.37	1.87	⇩ vs. WT
atg1Δ	1.10	0.63	⬆ vs. WT
atg1Δ + PE6	0.20	3.47	⇩ vs. WT<br>⇩ vs. WT + PE6<br>⇩ vs. *atg1Δ*

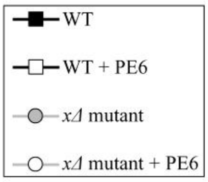

Supplementary Figure S9. Analysis of the Gompertz mortality function indicates that PE6 delays yeast chronological aging independently of presently known longevity-defining signaling pathways/protein kinases. Cells of the wild-type (WT) and indicated mutant strains were grown in the synthetic minimal YNB medium (0.67% Yeast Nitrogen Base without amino acids) initially containing 2% glucose, in the presence of 1.0% PE6 (ethanol was used as a vehicle at the final concentration of 5.0%) or in its absence (cells were subjected to ethanol-mock treatment). Survival curves shown in Fig. 5A were used to calculate the age-specific mortality rates (q_x), the Gompertz mortality rates (also known as mortality rate coefficient α) and the mortality rate doubling times (MRDT) for WT and mutant yeast populations cultured with or without 1.0% PE6. The values of q_x, α and MRDT were calculated as described in Materials and methods. Data for the mock-treated WT strain and for the WT strain cultured with PE6 are replicated in all graphs of this Supplementary Figure.

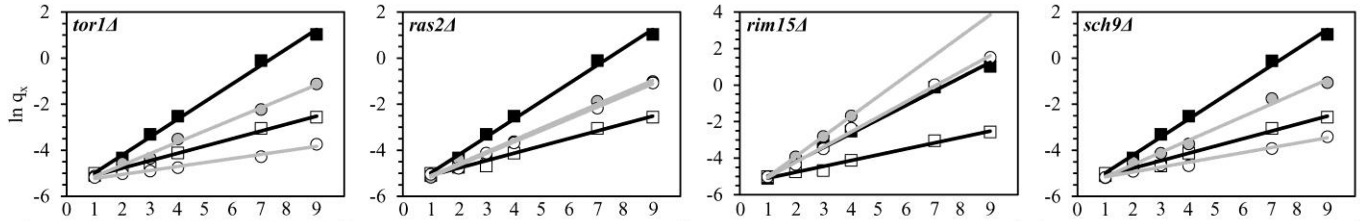

	α	MRDT	Rate of aging
WT	0.78	0.89	
WT + PE8	0.32	2.17	⇩ vs. WT
tor1Δ	0.50	1.39	⇩ vs. WT
tor1Δ + PE8	0.17	4.08	⇩ vs. WT<br>⇩ vs. WT + PE8<br>⇩ vs. *tor1Δ*

	α	MRDT	Rate of aging
WT	0.78	0.89	
WT + PE8	0.32	2.17	⇩ vs. WT
ras2Δ	0.52	1.33	⇩ vs. WT
ras2Δ + PE8	0.50	1.39	⇩ vs. WT<br>⬆ vs. WT + PE8<br>⇔ vs. *ras2Δ*

	α	MRDT	Rate of aging
WT	0.78	0.89	
WT + PE8	0.32	2.17	⇩ vs. WT
rim15Δ	1.11	0.62	⬆ vs. WT
rim15Δ + PE8	0.83	0.83	⬆ vs. WT<br>⬆ vs. WT + PE8<br>⇩ vs. *rim15Δ*

	α	MRDT	Rate of aging
WT	0.78	0.89	
WT + PE8	0.32	2.17	⇩ vs. WT
sch9Δ	0.53	1.31	⇩ vs. WT
sch9Δ + PE8	0.21	3.30	⇩ vs. WT<br>⇩ vs. WT + PE8<br>⇩ vs. *sch9Δ*

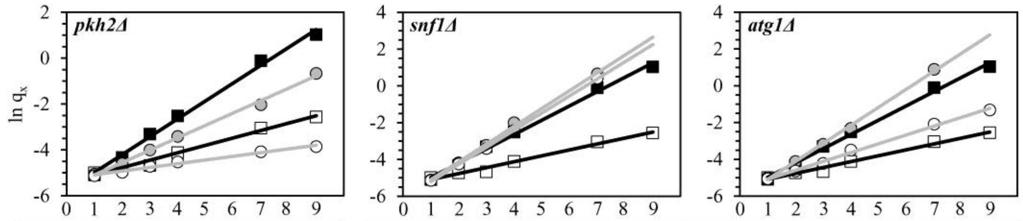

	α	MRDT	Rate of aging
WT	0.78	0.89	
WT + PE8	0.32	2.17	⇩ vs. WT
pkh2Δ	0.54	0.78	⇩ vs. WT
pkh2Δ + PE8	0.16	4.33	⇩ vs. WT<br>⇩ vs. WT + PE8<br>⇩ vs. *pkh2Δ*

	α	MRDT	Rate of aging
WT	0.78	0.89	
WT + PE8	0.32	2.17	⇩ vs. WT
snf1Δ	0.97	0.71	⬆ vs. WT
snf1Δ + PE8	0.93	0.75	⬆ vs. WT<br>⬆ vs. WT + PE8<br>⇔ vs. *snf1Δ*

	α	MRDT	Rate of aging
WT	0.78	0.89	
WT + PE8	0.32	2.17	⇩ vs. WT
atg1Δ	0.98	0.70	⬆ vs. WT
atg1Δ + PE8	0.48	1.44	⇩ vs. WT<br>⬆ vs. WT + PE8<br>⇩ vs. *atg1Δ*

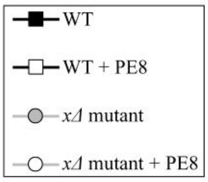

Supplementary Figure S10. Analysis of the Gompertz mortality function indicates that PE8 delays yeast chronological aging by attenuating the inhibitory effect of PKA on SNF1. Cells of the wild-type (WT) and indicated mutant strains were grown in the synthetic minimal YNB medium (0.67% Yeast Nitrogen Base without amino acids) initially containing 2% glucose, in the presence of 0.3% PE8 (ethanol was used as a vehicle at the final concentration of 1.5%) or in its absence (cells were subjected to ethanol-mock treatment). Survival curves shown in Fig. 6A were used to calculate the age-specific mortality rates (q_x), the Gompertz mortality rates (also known as mortality rate coefficient α) and the mortality rate doubling times (MRDT) for WT and mutant yeast populations cultured with or without 0.3% PE8. The values of q_x, α and MRDT were calculated as described in Materials and methods. Data for the mock-treated WT strain and for the WT strain cultured with PE8 are replicated in all graphs of this Supplementary Figure.

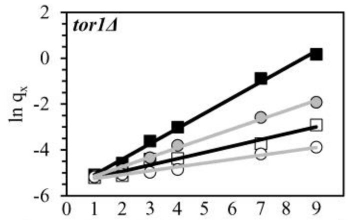

	α	MR DT	Rate of aging
WT	0.67	1.03	
WT + PE12	0.28	2.48	⇩ vs. WT
tor1Δ	0.41	1.69	⇩ vs. WT
tor1Δ + PE12	0.17	4.08	⇩ vs. WT<br>⇩ vs. WT + PE12<br>⇩ vs. *tor1Δ*

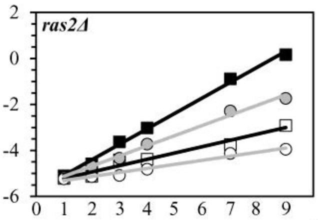

	α	MR DT	Rate of aging
WT	0.67	1.03	
WT + PE12	0.28	2.48	⇩ vs. WT
ras2Δ	0.44	1.58	⇩ vs. WT
ras2Δ + PE12	0.18	3.85	⇩ vs. WT<br>⇩ vs. WT + PE12<br>⇩ vs. *ras2Δ*

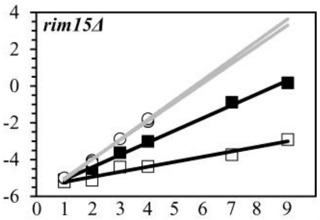

	α	MR DT	Rate of aging
WT	0.67	1.03	
WT + PE12	0.28	2.48	⇩ vs. WT
rim15Δ	1.04	0.67	⬆ vs. WT
rim15Δ + PE12	1.09	0.64	⬆ vs. WT<br>⬆ vs. WT + PE12<br>⇔ vs. *rim15Δ*

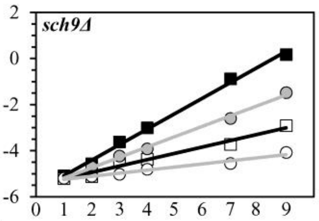

	α	MR DT	Rate of aging
WT	0.67	1.03	
WT + PE12	0.28	2.48	⇩ vs. WT
sch9Δ	0.45	1.54	⇩ vs. WT
sch9Δ + PE12	0.14	4.95	⇩ vs. WT<br>⇩ vs. WT + PE12<br>⇩ vs. *sch9Δ*

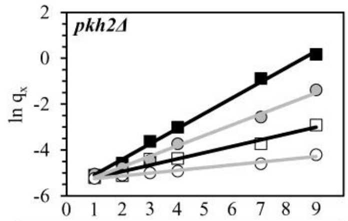

	α	MR DT	Rate of aging
WT	0.67	1.03	
WT + PE12	0.28	2.48	⇩ vs. WT
pkh2Δ	0.46	1.51	⇩ vs. WT
pkh2Δ + PE12	0.12	5.78	⇩ vs. WT<br>⇩ vs. WT + PE12<br>⇩ vs. *pkh2Δ*

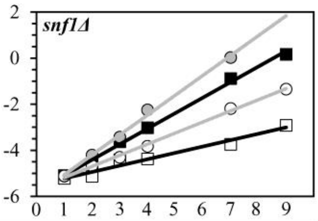

	α	MR DT	Rate of aging
WT	0.67	1.03	
WT + PE12	0.28	2.48	⇩ vs. WT
snf1Δ	0.86	0.81	⬆ vs. WT
snf1Δ + PE12	0.48	1.44	⇩ vs. WT<br>⬆ vs. WT + PE12<br>⇩ vs. *snf1Δ*

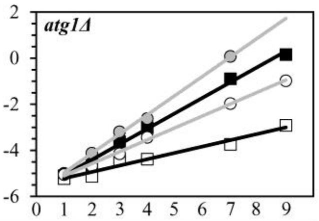

	α	MR DT	Rate of aging
WT	0.67	1.03	
WT + PE12	0.28	2.48	⇩ vs. WT
atg1Δ	0.84	0.83	⬆ vs. WT
atg1Δ + PE12	0.52	1.33	⇩ vs. WT<br>⬆ vs. WT + PE12<br>⇩ vs. *atg1Δ*

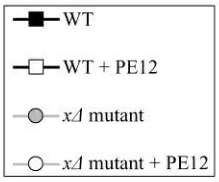

Supplementary Figure S11. Analysis of the Gompertz mortality function indicates that PE12 delays yeast chronological aging by stimulating Rim15. Cells of the wild-type (WT) and indicated mutant strains were grown in the synthetic minimal YNB medium (0.67% Yeast Nitrogen Base without amino acids) initially containing 2% glucose, in the presence of 0.1% PE12 (ethanol was used as a vehicle at the final concentration of 0.5%) or in its absence (cells were subjected to ethanol-mock treatment). Survival curves shown in Fig. 7A were used to calculate the age-specific mortality rates (q_x), the Gompertz mortality rates (also known as mortality rate coefficient α) and the mortality rate doubling times (MRDT) for WT and mutant yeast populations cultured with or without 0.1% PE12. The values of q_x, α and MRDT were calculated as described in Materials and methods. Data for the mock-treated WT strain are replicated in all graphs of this Supplementary Figure and in all graphs of Supplementary Figure S12. Data for each of the mock-treated mutant strains presented in this Supplementary Figure are replicated in the corresponding graphs of Supplementary Figure S12. Data for the WT strain cultured with PE12 are replicated in all graphs of this Supplementary Figure.

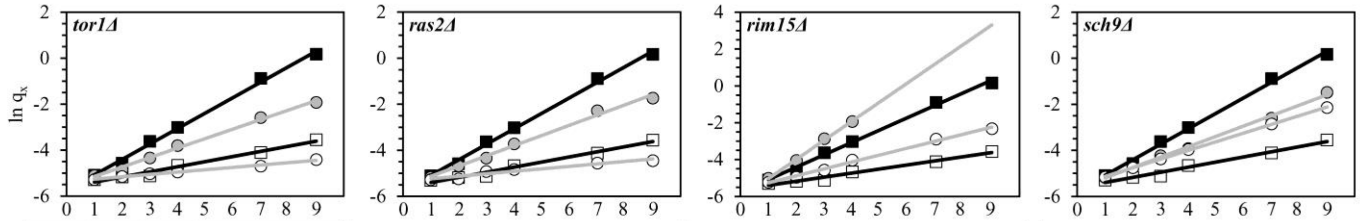

	α	MR DT	Rate of aging
WT	0.67	1.03	
WT + PE21	0.22	3.15	⇩ vs. WT
tor1Δ	0.41	1.69	⇩ vs. WT
tor1Δ + PE21	0.10	6.93	⇩ vs. WT<br>⇩ vs. WT + PE21<br>⇩ vs. *tor1Δ*

	α	MR DT	Rate of aging
WT	0.67	1.03	
WT + PE21	0.22	3.15	⇩ vs. WT
ras2Δ	0.44	1.58	⇩ vs. WT
ras2Δ + PE21	0.11	6.3	⇩ vs. WT<br>⇩ vs. WT + PE21<br>⇩ vs. *ras2Δ*

	α	MR DT	Rate of aging
WT	0.67	1.03	
WT + PE21	0.22	3.15	⇩ vs. WT
rim15Δ	1.04	0.67	⬆ vs. WT
rim15Δ + PE21	0.38	1.82	⇩ vs. WT<br>⬆ vs. WT + PE21<br>⇩ vs. *rim15Δ*

	α	MR DT	Rate of aging
WT	0.67	1.03	
WT + PE21	0.22	3.15	⇩ vs. WT
sch9Δ	0.45	1.54	⇩ vs. WT
sch9Δ + PE21	0.38	1.82	⇩ vs. WT<br>⬆ vs. WT + PE21<br>⇩ vs. *sch9Δ*

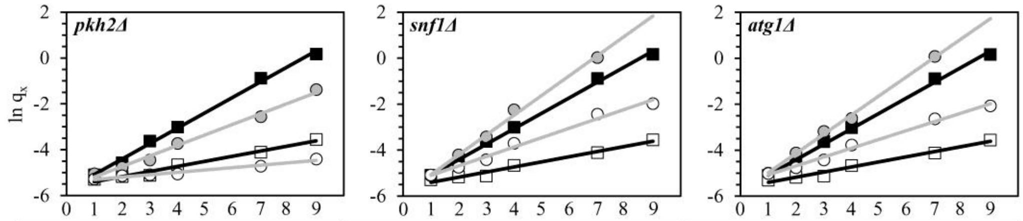

	α	MR DT	Rate of aging
WT	0.67	1.03	
WT + PE21	0.22	3.15	⇩ vs. WT
pkh2Δ	0.46	1.51	⇩ vs. WT
pkh2Δ + PE21	0.10	6.93	⇩ vs. WT<br>⇩ vs. WT + PE21<br>⇩ vs. *pkh2Δ*

	α	MR DT	Rate of aging
WT	0.67	1.03	
WT + PE21	0.22	3.15	⇩ vs. WT
snf1Δ	0.86	0.81	⬆ vs. WT
snf1Δ + PE21	0.41	1.69	⇩ vs. WT<br>⬆ vs. WT + PE21<br>⇩ vs. *snf1Δ*

	α	MR DT	Rate of aging
WT	0.67	1.03	
WT + PE21	0.22	3.15	⇩ vs. WT
atg1Δ	0.84	0.83	⬆ vs. WT
atg1Δ + PE21	0.39	1.78	⇩ vs. WT<br>⬆ vs. WT + PE21<br>⇩ vs. *atg1Δ*

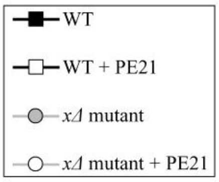

Supplementary Figure S12. Analysis of the Gompertz mortality function indicates that PE21 delays yeast chronological aging by inhibiting a PKH1/2-sensitive form of Sch9. Cells of the wild-type (WT) and indicated mutant strains were grown in the synthetic minimal YNB medium (0.67% Yeast Nitrogen Base without amino acids) initially containing 2% glucose, in the presence of 0.1% PE21 (ethanol was used as a vehicle at the final concentration of 0.5%) or in its absence (cells were subjected to ethanol-mock treatment). Survival curves shown in Fig. 8A were used to calculate the age-specific mortality rates (q_x), the Gompertz mortality rates (also known as mortality rate coefficient α) and the mortality rate doubling times (MRDT) for WT and mutant yeast populations cultured with or without 0.1% PE21. The values of q_x, α and MRDT were calculated as described in Materials and methods. Data for the mock-treated WT strain are replicated in all graphs of this Supplementary Figure and in all graphs of Supplementary Figure S11. Data for each of the mock-treated mutant strains presented in this Supplementary Figure are replicated in the corresponding graphs of Supplementary Figure S11. Data for the WT strain cultured with PE21 are replicated in all graphs of this Supplementary Figure.
